# Development and validation of the Durham Risk Score for estimating suicide attempt risk: A prospective cohort analysis

**DOI:** 10.1371/journal.pmed.1003713

**Published:** 2021-08-05

**Authors:** Nathan A. Kimbrel, Jean C. Beckham, Patrick S. Calhoun, Bryann B. DeBeer, Terence M. Keane, Daniel J. Lee, Brian P. Marx, Eric C. Meyer, Sandra B. Morissette, Eric B. Elbogen

**Affiliations:** 1 Durham Veterans Affairs (VA) Health Care System, Durham, North Carolina, United States of America; 2 VA Mid-Atlantic Mental Illness Research, Education, and Clinical Center, Durham, North Carolina, United States of America; 3 VA Health Services Research and Development Center of Innovation to Accelerate Discovery and Practice Transformation, Durham, North Carolina, United States of America; 4 Department of Psychiatry and Behavioral Sciences, Duke University School of Medicine, Durham, North Carolina, United States of America; 5 Rocky Mountain Mental Illness Research, Education, and Clinical Center, Denver, Colorado, United States of America; 6 National Center for PTSD, Boston, Massachusetts, United States of America; 7 VA Boston Healthcare System, Boston, Massachusetts, United States of America; 8 Boston University School of Medicine, Boston, Massachusetts, United States of America; 9 Department of Rehabilitation Science and Technology, University of Pittsburgh, Pittsburgh, Pennsylvania, United States of America; 10 Department of Psychology, The University of Texas at San Antonio, San Antonio, Texas, United States of America; Harvard Medical School, UNITED STATES

## Abstract

**Background:**

Worldwide, nearly 800,000 individuals die by suicide each year; however, longitudinal prediction of suicide attempts remains a major challenge within the field of psychiatry. The objective of the present research was to develop and evaluate an evidence-based suicide attempt risk checklist [i.e., the Durham Risk Score (DRS)] to aid clinicians in the identification of individuals at risk for attempting suicide in the future.

**Methods and findings:**

Three prospective cohort studies, including a population-based study from the United States [i.e., the National Epidemiologic Survey on Alcohol and Related Conditions (NESARC) study] as well as 2 smaller US veteran cohorts [i.e., the Assessing and Reducing Post-Deployment Violence Risk (REHAB) and the Veterans After-Discharge Longitudinal Registry (VALOR) studies], were used to develop and validate the DRS. From a total sample size of 35,654 participants, 17,630 participants were selected to develop the checklist, whereas the remaining participants (*N* = 18,024) were used to validate it. The main outcome measure was future suicide attempts (i.e., actual suicide attempts that occurred after the baseline assessment during the 1- to 3-year follow-up period). Measure development began with a review of the extant literature to identify potential variables that had substantial empirical support as longitudinal predictors of suicide attempts and deaths. Next, receiver operating characteristic (ROC) curve analysis was utilized to identify variables from the literature review that uniquely contributed to the longitudinal prediction of suicide attempts in the development cohorts. We observed that the DRS was a robust prospective predictor of future suicide attempts in both the combined development (area under the curve [AUC] = 0.91) and validation (AUC = 0.92) cohorts. A concentration of risk analysis found that across all 35,654 participants, 82% of prospective suicide attempts occurred among individuals in the top 15% of DRS scores, whereas 27% occurred in the top 1%. The DRS also performed well among important subgroups, including women (AUC = 0.91), men (AUC = 0.93), Black (AUC = 0.92), White (AUC = 0.93), Hispanic (AUC = 0.89), veterans (AUC = 0.91), lower-income individuals (AUC = 0.90), younger adults (AUC = 0.88), and lesbian, gay, bisexual, transgender, and queer or questioning (LGBTQ) individuals (AUC = 0.88). The primary limitation of the present study was its its reliance on secondary data analyses to develop and validate the risk score.

**Conclusions:**

In this study, we observed that the DRS was a strong predictor of future suicide attempts in both the combined development (AUC = 0.91) and validation (AUC = 0.92) cohorts. It also demonstrated good utility in many important subgroups, including women, men, Black, White, Hispanic, veterans, lower-income individuals, younger adults, and LGBTQ individuals. We further observed that 82% of prospective suicide attempts occurred among individuals in the top 15% of DRS scores, whereas 27% occurred in the top 1%. Taken together, these findings suggest that the DRS represents a significant advancement in suicide risk prediction over traditional clinical assessment approaches. While more work is needed to independently validate the DRS in prospective studies and to identify the optimal methods to assess the constructs used to calculate the score, our findings suggest that the DRS is a promising new tool that has the potential to significantly enhance clinicians’ ability to identify individuals at risk for attempting suicide in the future.

## Abstract 2

Using data from three prospective cohorts in the US, Nathan Kimbrel and colleagues report on the development and validation of an evidence-based checklist to identify those at risk of future suicide attempts.

## Author summary

**Why was this study done?:**

- Nearly 800,000 individuals die by suicide each year worldwide; however, longitudinal prediction of suicide attempts remains a major challenge within the field of psychiatry.
- Current clinical risk instruments and assessments to detect risk for future suicide attempts lack sufficient diagnostic accuracy to guide treatment decisions.

**What did the researchers do and find?:**

- The goal of this study was to develop and evaluate a risk score (the Durham Risk Score or DRS) to aid clinicians in identifying individuals at risk for attempting suicide.
- Secondary analyses were conducted on 3 prospective cohort studies from the US (total sample size = 35,654 participants), including a large general population study and 2 smaller veteran cohorts.
- The risk score was a strong predictor of future suicide attempts in both the combined development (area under the curve [AUC] = 0.91) and validation (AUC = 0.92) cohorts. Moreover, 82% of prospective suicide attempts occurred among individuals in the top 15% of risk scores, whereas 27% occurred among individuals scoring in the top 1% of risk scores.
- The risk score also performed well among important subgroups, including women, men, Black, White, Hispanic, veterans, lower-income individuals, younger adults, and lesbian, gay, bisexual, transgender, and queer or questioning (LGBTQ) individuals.

**What do these findings mean?:**

- Our findings suggest that the DRS is a promising new tool that has the potential to enhance clinicians’ ability to identify individuals at risk for attempting suicide.
- The primary limitation of this work is its reliance on secondary data analyses to develop and validate the score.
- More work is needed to independently validate the DRS in prospective studies and to identify the optimal methods to assess each of the constructs used to calculate the score.

## Introduction

Suicide accounted for 793,000 deaths worldwide in 2016 and was the second leading cause of death among 15 to 29 year olds [1]. Moreover, within the US, age-adjusted suicide rates have increased by 33% since 1999 [2]. Unfortunately, prospective prediction of suicidal behavior remains a major challenge for the field of psychiatry [3]. For example, a 2017 meta-analysis of longitudinal risk factors for suicidal behavior found the overall weighted odds ratio (OR) for prospective predictors of suicide attempts to be 1.5 [3]. When diagnostic accuracy was examined, no risk factor category (including suicide screeners) had a weighted area under the curve (AUC) greater than 0.61 for the prediction of future suicide attempts [3]. Similarly, a 2019 study [4] designed to prospectively evaluate several of the most commonly used suicide attempt risk instruments in the US, including the Columbia-Suicide Severity Rating Scale (C-SSRS [5]; a widely used suicide risk assessment instrument recommended for use in drug trials [6]), the Self-Harm Behavior Questionnaire (SHBQ [7]), the Suicidal Behaviors Questionnaire-Revised (SBQ-R [8]), and the Beck Scale for Suicide Ideation (BSS [9]), found that none of these instruments had an AUC above 0.67 in relation to future suicide attempts [4]. Similarly, a 2018 study by Randall and colleagues [10] also found that the C-SSRS was only moderately accurate at predicting future attempts (AUC = 0.67) and death by suicide (AUC = 0.68) [10].

In England, Quinlivan and colleagues investigated the extent and type of suicide risk scales utilized by emergency department clinicians and mental health staff members from a stratified random sample of 32 hospitals and found that the most frequently used suicide risk assessment instruments were unvalidated, locally developed scales [11]. Indeed, 22 of 32 (68.8%) English hospitals included in this study used an unvalidated instrument to assess suicide risk, leading the authors to conclude that there is presently little consensus among clinicians and hospital systems regarding the best instrument to use to assess suicide risk [11]. In the remaining third of English hospitals included in the study, the SAD PERSONS scale (SPS) [12] emerged as the most frequently used standardized approach to suicide risk assessment [11]. Unfortunately, recent studies have found that the AUC for the SPS for prediction of future suicide attempts is not better than chance (AUC = 0.51 to 0.57) [13,14]. Two other similarly structured and frequently used clinical risk approaches, including the Manchester Self-Harm Rule [15] and the ReACT Self-Harm Rule [16], performed better (AUC = 0.71 for both [13]), but still well below the level of discrimination typically recommended for clinical decision-making (i.e., AUC ≥0.90). While discouraging, these findings are consistent with a recent systematic review and meta-analysis of currently available suicide risk instruments including (among others) the C-SSRS [5], BSS [9], SPS [12], the Manchester Self-Harm Rule [15], and the ReACT Self-Harm Rule [16] that concluded that there is presently “… no scientific support for the use of suicide risk instruments for predicting suicidal acts” [17].

Given such findings, it is perhaps not surprising that the American Psychiatric Association’s (APA) Practice Guideline for the Assessment and Treatment of Patients with Suicidal Behaviors [18] recommends that psychiatrists utilize their clinical judgment to estimate patients’ overall level of suicide risk based on a comprehensive psychiatric evaluation, rather than relying on a standardized instrument to estimate suicide risk. The guideline further indicates that psychiatrists should consider no less than 70 different risk and protective factors when attempting to estimate patients’ suicide risk, including history of suicidal thoughts/behaviors (5 factors), psychiatric diagnoses (8 factors), physical illnesses (12 factors), psychosocial features (6 factors), childhood traumas (2 factors), genetic and familial effects (2 factors), psychological features (12 factors), cognitive features (4 factors), demographic features (6 factors), additional features (3 factors), and protective factors (10 factors) [18].

Regrettably, there is little reason to believe that clinician prediction is more accurate at predicting future suicidal behavior than structured assessments [10,19]. For example, Randall and colleagues [10] examined the accuracy of clinician prediction of suicide risk and found that clinician assessment was also only moderately accurate at predicting future suicide attempts (AUC = 0.73). Moreover, clinician prediction of future death by suicide was no better than chance (AUC = 0.55; 95% confidence interval [CI]: 0.36 to 0.73) [10]. These findings are consistent with a 2019 meta-analysis conducted by Woodford and colleagues [19] that evaluated the accuracy of clinician prediction in relation to future self-harm (note that the term “self-harm” encompasses both suicidal and nonsuicidal self-injury [NSSI]). This meta-analysis (which did not include the study by Randall and colleagues [10] cited above) estimated sensitivity for clinician prediction of future self-harm to be 0.31 [19], indicating that clinician prediction in the included studies failed to identify 69% of the individuals who went on to engage in future self-harm. While specificity (0.85) for clinician prediction of self-harm was markedly better than sensitivity, overall classification remained poor. Woodford and colleagues [19] did not report the AUC value for clinician prediction of future self-harm in their meta-analysis; however, in preparation for the present work, we utilized Idrees and colleagues’ [20] approach to calculate AUC from the classification data provided by Woodford and colleagues [19], which included 1,685 true positives (TPs), 5,996 false positives (FPs), 1,556 false negatives (FNs), and 13,262 true negatives (TNs). This calculation revealed that the AUC value for clinician prediction for future self-harm across the 22,499 cases examined by Woodford and colleagues [19] was 0.60 (where AUC = (1/2) * [(TP/(TP+FN)) + (TN/(TN+FP))]. Thus, we concur with Woodford and colleagues’ conclusion that clinician estimation of future self-harm is too inaccurate to be clinically useful [19].

As a result of concerns over the poor diagnostic accuracy of both clinician prediction [10,19] and existing clinical suicide risk assessments [3–17], a number of statistically driven suicide risk algorithms based on electronic health record (EHR) data have been developed in recent years and are already showing substantial promise [21–24]; however, such approaches have also been criticized for having limited practical utility [24]. In addition to problems related to low positive predictive values (PPVs) [10], such models also have pragmatic shortcomings, such as (1) not being available for individuals outside the healthcare systems where they were originally developed; (2) not being able to be applied to first-time patients or patients who do not meet certain criteria (e.g., a history of mental health appointments in the EHR); (3) being impractical for clinicians to calculate on their own; and (4) being difficult for clinicians to interpret because scores are often derived from machine learning approaches that rely on hidden layers, nonlinear models, and complex higher-order interactions. Thus, while machine learning–based algorithms derived from EHR data appear to substantially outperform clinician prediction and traditional clinical assessment approaches in terms of diagnostic accuracy, they also have a number of pragmatic shortcomings that potentially limit their usefulness for practicing clinicians.

**Fig 1:**
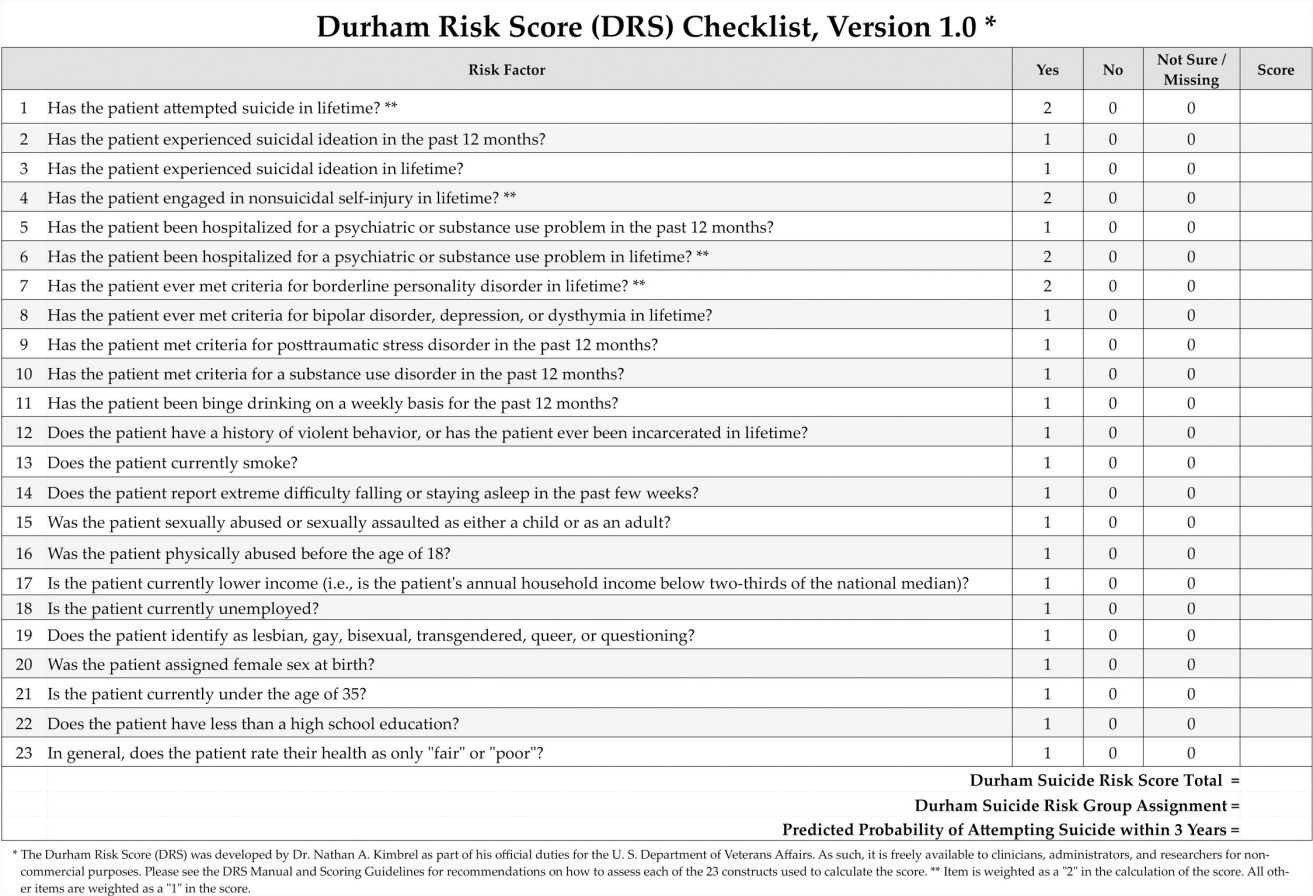
The DRS Checklist, Version 1.0. DRS, Durham Risk Score.

Thus, there remains a pressing need for a risk assessment tool capable of helping clinicians to accurately identify individuals at risk for attempting suicide in the future. The Durham Risk Score (DRS; **Fig 1**) is a suicide attempt risk checklist developed using both rational and quantitative methods to meet this specific need. This report describes the initial development and validation of the DRS, including its utility in predicting future suicide attempts over a 1- to 3-year period across a large and diverse cohort of participants from the US [25–27]. In creating this measure, our goal was to create a suicide risk calculator similar in nature to the well-known Framingham Risk Score and pooled cohort equations that are widely used to screen individuals for 10-year risk of cardiovascular disease [28]. We hypothesized that by combining a broad array of empirically supported risk factors for suicidal behavior [3–18,21–24,26,27,29–43] into a clinical checklist that we could significantly enhance clinicians’ ability to identify individuals at risk for attempting suicide in the future.

## Methods

### Participants

#### National Epidemiologic Survey on Alcohol and Related Conditions study

The National Epidemiologic Survey on Alcohol and Related Conditions (NESARC) study [25,44,45] is a large, longitudinal general population study conducted by the National Institute on Alcohol Abuse and Alcoholism in the US. The initial NESARC study included a nationally representative sample of 43,093 participants assessed for a wide array of psychiatric and substance use issues in 2001 to 2002 [25]. Wave 2 occurred 3 years later and included follow-up interviews with 34,653 of the participants from Wave 1 (see Grant and colleagues [25,44,45] for additional details regarding study procedures for the NESARC project). The current analyses were restricted to the 34,641 NESARC participants who participated in Waves 1 and 2 and had follow-up suicide attempt data available from Wave 2. The random selection procedure from the IBM SPSS Statistics 24 software package was used to generate 4 random subsets of participants from the NESARC dataset, including 2 for development [NESARC 1 (*N* = 8,872) and NESARC 2 (*N* = 8,525)] and 2 for validation [NESARC 3 (*N* = 8,516) and NESARC 4 (*N* = 8,728), see **Table 1** for sample characteristics]. Sampling was performed without replacement to ensure that each case was not selected more than once. Note that the 4 subsets of participants from NESARC did not differ by rate of prospective suicide attempts, *p* = 0.973; lifetime suicide attempts, *p* = 0.729; gender, *p* = 0.541; age, *p* = 0.448; race, *p* = 0.814; sexual orientation, *p* = 0.839; education, *p* = 0.343; income, *p* = 0.67; or employment status, *p* = 0.923. See Grant and colleagues [25,44,45] for additional details regarding study procedures for the NESARC study.

**Table 1:** Descriptive statistics for the longitudinal samples used to develop and validate the DRS.

	NESARC 1	NESARC 2	REHAB	NESARC 3	NESARC 4	VALOR
Key reference(s)	Grant et al. [25,44–45]	Grant et al. [25,44–45]	Elbogen et al. [46] and Adkisson et al. [26]	Grant et al. [25,44–45]	Grant et al. [25,44–45]	Rosen et al. [47] and Lee et al. [27]
Study name/acronym	NESARC	NESARC	REHAB	NESARC	NESARC	VALOR
Purpose	Development	Development	Development	Validation	Validation	Validation
Participants	8,872	8,525	233	8,516	8,728	780
Approximate follow-up period in years	2–3	2–3	1	2–3	2–3	2
Age mean	46.1	45.9	40.4	45.9	46.0	36.9
Age SD	17.4	17.4	10.6	17.3	17.4	9.7
Female	5,146 (58%)	4,971 (58.3%)	39 (16.7%)	4,959 (58.2%)	5,003 (57.3%)	405 (51.9%)
White	5,233 (59%)	4,982 (58.4%)	86 (36.9%)	4,885 (57.4%)	5,063 (58%)	637 (81.7%)
Black/African-American	1,666 (18.8%)	1,620 (19%)	127 (54.5%)	1,641 (19.3%)	1,649 (18.9%)	111 (14.2%)
Hispanic/Latino ethnicity	1,580 (17.8%)	1,563 (18.3%)	11 (4.7%)	1,596 (18.7%)	1,616 (18.5%)	91 (11.7%)
Military veteran	459 (5.2%)	430 (5%)	233 (100%)	442 (5.2%)	515 (5.9%)	780 (100%)
History of psychiatric disorders	4,679 (52.7%)	4,459 (52.3%)	165 (69.5%)	4,411 (51.8%)	4,590 (52.6%)	599 (76.8%)
History of suicide attempts	224 (2.5%)	213 (2.5%)	11 (4.7%)	234 (2.7%)	229 (2.6%)	634 (18.7%)
Prospective attempts during study	62 (0.7%)	57 (0.7%)	10 (4.3%)	62 (0.7%)	60 (0.7%)	37 (4.7%)
DRS items assessed	21 (91%)	21 (91%)	18 (78%)	21 (91%)	21 (91%)	15 (65%)
DRS minimum	0	0	0	0	0	0
DRS maximum	20	22	13	20	20	15
DRS SD	2.5	2.5	2.6	2.6	2.5	3.0
DRS mean	3.2	3.3	3.9	3.3	3.3	5.8
DRS median	3	3	3	3	3	6
DRS mode	2	2	3	2	2	3
DRS skewness	1.8	1.9	1.1	1.8	1.8	0.4
DRS kurtosis	5.2	5.4	1	5	5.3	−0.4

*Lifetime suicidal ideation was not directly assessed in VALOR; however, consistent with scoring procedures, participants with a history of attempts or current ideation were scored as also having lifetime ideation; thus, only 15 of the 23 (i.e., 65%) total DRS items were directly assessed as part of the VALOR study.

DRS, Durham Risk Score; NESARC, National Epidemiologic Survey on Alcohol and Related Conditions; REHAB, Assessing and Reducing Post-Deployment Violence Risk; SD, standard deviation; VALOR, Veterans After-Discharge Longitudinal Registry.

#### Assessing and Reducing Post-Deployment Violence Risk study

The Assessing and Reducing Post-Deployment Violence Risk (REHAB) sample was comprised of Iraq/Afghanistan-era veterans from the US who participated in a 1-year longitudinal study entitled “Assessing and Reducing Post-Deployment Violence Risk” that focused on examining the association between post-traumatic stress disorder (PTSD), traumatic brain injury (TBI), and violence [26,46].To be eligible for the present analyses, participants had to have no history of post-deployment suicide attempts at the time of the baseline assessment as well as follow-up suicide attempt data available for analysis. The former inclusion criteria were used to ensure that all prospective suicide attempts reported at the 6- and 12-month follow-up assessments truly represented new instances of suicide attempts, as this study relied exclusively on self-report to assess suicide attempts. Additional details regarding this study’s methodology can be found in Elbogen and colleagues [46] and Adkisson and colleagues [26].

#### Veterans After-Discharge Longitudinal Registry study

The Veterans After-Discharge Longitudinal Registry (VALOR) sample was comprised of US veterans who participated in the VALOR study [27,47], a 2-year longitudinal study of Iraq/Afghanistan-era veterans. Analyses were limited to participating veterans (*N* = 780) with complete baseline data and follow-up suicide attempt data available for analysis. Further details regarding this study’s methodology can be found in Rosen and colleagues [47] and Lee and colleagues [27].

### Main outcome variable

The present analyses focused on the prediction of future suicide attempts (as opposed to death by suicide) for several reasons. First, death by suicide is an extremely rare event. In the US, the age-adjusted rate of suicide was 13.9/100,000 in 2019 [2]. Suicide attempts are far more common than suicide deaths [2,31,32] and are among the strongest known predictors of death by suicide [3,31,32]. Indeed, Olfson and colleagues [32] found that 1.6% of individuals who attempted suicide died by suicide within 12 months, whereas 3.9% died by suicide within 5 years. Suicide attempts are also routinely assessed in high-quality longitudinal datasets, whereas there are few, if any, longitudinal research databases with sufficiently large samples sizes to study death by suicide that also contain high-quality, systematically assessed data on established predictors of suicidal behavior collected via rigorous research-based assessments. Of note, Belsher and colleagues [24] recently recommended that future suicide risk models target more common outcomes, including suicide attempts specifically, to develop better performing models of suicide risk following their review of existing suicide risk models. It is also important to recognize that suicide attempts are highly serious events in their own right. As noted by the World Health Organization [48], “Suicide attempts result in a significant social and economic burden for communities due to the utilization of health services to treat the injury, the psychological and social impact of the behaviour on the individual and his/her associates and, occasionally, the long-term disability due to the injury.”

#### Assessment of suicide attempts

Prospective suicide attempts were assessed by trained interviewers in the NESARC study during the Low Mood portion of the interview with the following question: “During that time since your LAST interview when (your mood was at its lowest/you enjoyed or cared least about things), did you attempt suicide?” Thus, for the vast majority of participants included in the present analyses, the main outcome variable was assessed by an interviewer who was explicitly trained to only record new instances of suicide attempts that occurred after the initial NESARC baseline assessment.

Similarly, in the VALOR sample, the Self-Injurious Thoughts and Behaviors Interview (SITBI) [49] was administered at the 2-year follow-up by a trained interviewer who specifically focused on identifying new instances of suicide attempts that had occurred since the time of the baseline assessment. Specifically, Project VALOR participants were asked the following question in relation to the 2-year time period following the baseline assessment: “Have you ever made an actual attempt to kill yourself in which you had at least some intent to die?” Participants’ EHRs were also reviewed for instances of suicide attempts and/or death by suicide as part of Project VALOR. Further details regarding these procedures can be found in Lee and colleagues [27] and Rosen and colleagues [47].

Finally, in the REHAB sample, suicide attempts were assessed via self-report with a study-specific instrument designed to assess pre-deployment suicide attempts, deployment-based suicide attempts, and post-deployment suicide attempts separately [26]. Because this was the only study included in the present analyses that relied exclusively on self-report to assess prospective suicide attempts, veterans who reported 1 or more post-deployment suicide attempts at the time of the baseline assessment were excluded from the present analyses to ensure that any new instances of post-deployment suicide attempts reported at the 6- and 12-month follow-up assessments truly reflected new occurrences of suicide attempts and were not the result of a reporting error.

### Overview of the analysis plan

The primary analyses underlying the development and validation of the DRS began in April 2018 and ended in July 2020 and were conducted under research protocols approved by the Institutional Review Boards of the Durham Veterans Affairs Health Care System, Duke University School of Medicine, and the VA Boston Healthcare System. Additional analyses requested by reviewers during the peer review process were conducted from March 2021 to April 2021. While a written prospective analysis plan was not developed prior to initiating work on this project, a systematic approach was used to develop and validate the DRS. Specifically, measure development began with a review of the extant literature on risk factors for suicidal behavior [3–18,21–24,26,27,29–43]. After identifying and ranking a wide array of potential longitudinal predictors of death by suicide and suicide attempts from the literature, secondary data analyses were conducted to develop the DRS in the development samples (i.e., NESARC 1, NESARC 2, and REHAB; combined *N* = 17,630). It was then tested in the validation samples (i.e., NESARC 3, NESARC 4, and VALOR; combined *N* = 18,024) to determine if it continued to be predictive in separate cohorts of similar size and composition. This study is reported as per the Transparent Reporting of a multivariable prediction model for Individual Prognosis Or Diagnosis (TRIPOD) reporting guideline (see **S1 TRIPOD Checklist**).

### Development of the Durham Risk Score

Because our primary goal was to develop a suicide attempt risk checklist that could be used by clinicians to reliably discriminate high-risk patients from low-risk patients, receiver operating characteristic (ROC) curve analysis was the primary statistical approach used to develop the DRS in the development samples. Logistic regression was also utilized to help guide variable selection procedures in some instances. ROC curves, correlation matrices, and chi-squared tests were used to evaluate bivariate associations and to identify optimal cut points or iterations of variables that were maximally predictive of suicide attempts.

We elected to split the NESARC sample into 4 smaller samples to ensure that we would have (1) 2 large datasets with which to conduct the initial development work in; and (2) 2 large datasets of similar size and composition in which to test the performance of the final selected model. That is, consistent with standard holdout cross-validation approaches that utilize a training dataset (T_*tr*_) and a validation dataset (T_*v*_) to avoid overfitting due to limiting the development sample to a single dataset, we utilized 2 large, randomly selected subsets of NESARC participants to develop the DRS. A third sample (REHAB), which was smaller, collected independently, and comprised entirely of veterans (many of whom had psychiatric disorders), was also included in the development phase to further protect against overfitting and to increase generalizability of findings. Thus, from a total sample size of 35,654 participants, 17,630 participants (including NESARC 1, NESARC 2, and REHAB) were utilized to develop the DRS, whereas the remaining samples (NESARC 3, NESARC 4, and VALOR; combined *N* = 18,024) were held out to test the performance of the DRS in testing datasets (T*t*) of similar size and composition. **Table 1** provides descriptive statistics for each of the samples included in the present analyses.

Consistent with recommendations for building appropriate and stable predictive models [50,51], independent variable selection was guided by theory [29,30], prior empirical investigations [3–5,7–18,21–24,26,27,29–43], clinical considerations [3–18,21–24,29–31,48], univariate and bivariate statistical analyses, and consideration of multicollinearity among independent variables. Accordingly, independent variable selection and screening began with a review of the relevant literature concerning risk factors for suicidal behavior [3–5,7–18,21–24,26,27,29–43]. An a priori decision was made to prioritize variables that had particularly strong empirical support as longitudinal risk factors in the literature (e.g., recent psychiatric hospitalization)—even if their effects were less pronounced in our specific samples—in hopes of increasing the stability and replicability of the checklist in future work.

To simplify quantification of the empirical evidence, we relied on Franklin and colleagues’ [3] meta-analysis, which, in our opinion, was the most comprehensive work on this subject available at the time of the analyses. The top 10 broad risk categories for suicide deaths and suicide attempts were assigned scores from 1 to 10, where a score of 10 was assigned to the broad risk categories most strongly associated with suicide deaths and attempts. In addition, the top 5 predictors of suicide deaths and suicide attempts identified in this meta-analysis were also assigned scores from 6 to 10. Thus, potential evidence scores ranged from 0 to 40 (see **Table A in S1 File**). **Table 2** provides the empirical evidence score that we assigned to each of the variables based on the findings from Franklin and colleagues [3] as well as the potential impact of each variable’s entry into the model on the cumulative AUC value for different iterations of the DRS across the 3 development samples.

**Table 2:** Empirical evidence scores and impact on cumulative AUC values for each of the DRS variables across the 3 development samples.

Entry order	Variable	Broad risk category	Evidence for category for suicide death [3]*	Evidence for category for attempts [3]*	Evidence for variable for suicide death [3]*	Evidence for variable for attempts [3]*	Total evidence score*	NESARC 1 cumulative AUC	NESARC 2 cumulative AUC	REHAB cumulative AUC
1	Suicide attempt—lifetime	Prior SITBIs	7	10	9	9	35	0.70	0.62	0.53
2	Hospitalization (past year)	Treatment history	10	5	10	6	31	0.70	0.63	0.53
3	Hospitalization—lifetime	Treatment history	10	5	10	6	31	0.74	0.67	0.64
4	NSSI (lifetime)	Prior SITBIs	7	10	0	10	27	0.79	0.78	0.69
5	Suicidal ideation (past year)	Prior SITBIs	7	10	8	0	25	0.79	0.78	0.78
6	Suicidal ideation (lifetime)	Prior SITBIs	7	10	8	0	25	0.82	0.78	0.80
7	BPD (lifetime)	Psychopathology	8	8	0	7	23	0.90	0.84	0.80
8	Unemployed	Social factors	6	1	6	0	13	0.90	0.83	0.80
9	Poor perceived health	Physical illness	5	6	0	0	11	0.90	0.84	0.80
10	Lower income	Demographics	3	0	7	0	10	0.90	0.84	0.80
11	Child physical abuse (lifetime)	Social factors	6	1	0	0	7	0.91	0.86	0.85
12	Sexual abuse/assault (lifetime)	Social factors	6	1	0	0	7	0.92	0.87	0.85
13	Severe sleep problems (past year)	Internalizing	2	4	0	0	6	0.92	0.87	0.86
14	PTSD (past year)	Internalizing	2	4	0	0	6	0.92	0.88	0.86
15	Mood disorder (lifetime)	Internalizing	2	4	0	0	6	0.92	0.87	0.89
16	Violence/incarceration (lifetime)	Externalizing	4	0	0	0	4	0.92	0.87	0.86
17	Weekly binges (past year)	Externalizing	4	0	0	0	4	0.92	0.86	0.87
18	Current smoker	Externalizing	4	0	0	0	4	0.92	0.86	0.88
19	Substance use disorder (past year)	Externalizing	4	0	0	0	4	0.91	0.86	0.89
20	Younger than 35	Demographics	3	0	0	0	3	0.91	0.87	0.89
21	Less than HS education	Demographics	3	0	0	0	3	0.92	0.87	0.89
22	Sexual minority	Demographics	3	0	0	0	3	0.92	0.87	0.89
23	Female sex at birth	Demographics	3	0	0	0	3	0.92	0.87	0.90
24	Suicide attempt—lifetime	Prior SITBIs	7	10	9	9	35	0.92	0.87	0.89
25	Hospitalization—lifetime	Treatment history	10	5	10	6	31	0.92	0.87	0.88
26	NSSI (lifetime)	Prior SITBIs	7	10	0	10	27	0.92	0.88	0.88
27	BPD (lifetime)	Psychopathology	8	8	0	7	23	0.93	0.89	0.88

*The top 10 broad risk categories for suicide deaths and suicide attempts from [3] were assigned scores from 1 to 10, where a score of 10 was assigned to the category most strongly associated with suicide deaths and attempts. Similarly, the top 5 predictors of suicide deaths and suicide attempts were assigned scores from 6 to 10. Thus, total evidence scores could potentially range from 0 to 40.

AUC, area under the curve; BPD, borderline personality disorder; DRS, Durham Risk Score; HS, high school; NESARC, National Epidemiologic Survey on Alcohol and Related Conditions; NSSI, nonsuicidal self-injury; PTSD, post-traumatic stress disorder; REHAB, Assessing and Reducing Post-Deployment Violence Risk; SITBI, Self-Injurious Thoughts and Behaviors Interview.

As can be seen in **Table 2** and **Table A in S1 File**, a history of prior suicide attempts was the variable with the highest total empirical evidence score based on this approach (total empirical evidence score = 35; mean AUC = 0.62), whereas psychosis/schizophrenia was the lowest scoring variable (total empirical evidence score = 1; mean AUC = 0.52) that was considered. As can be seen in **Fig A in S1 File**, a statistically significant positive correlation (*r* = 0.37, *p* < 0.001) was observed between the total empirical evidence score and the mean bivariate AUC value for each construct considered across the 3 development samples, providing support for our general (albeit simplistic) approach to quantifying the empirical evidence for the variables considered.

To ensure that scoring and interpretation remained as simple as possible (i.e., to ensure that higher scores would equal higher risk), an a priori decision was also made to only include dichotomous risk factors with obvious main effects. Thus, protective factors, risk factors that only had effects in the presence of other variables (e.g., through interactions), and scaled risk factors were excluded as potential predictors (although in several cases we were able to successfully dichotomize items collected on a scale, e.g., sleep problems and perceived health). Additionally, consistent with Babyak’s recommendations [50], overlapping constructs were aggregated in many instances to increase model stability and reduce the number of variables included in the checklist. As a result, composite variables were created for “mood disorders,” “substance use disorders,” “violence/incarceration,” “sexual abuse/sexual assault,” and “lesbian, gay, bisexual, transgender, and queer or questioning (LGBTQ)”.

An iterative, sequential approach to model building was taken whereby variables expected to have strong and pronounced effects on future risk for suicide based on the extant literature (e.g., prior suicide attempts, hospitalization, NSSI, and suicidal ideation) [3] were entered before variables with less empirical support (e.g., demographic predictors). We began by calculating ROC curves for each of the potential predictors across the 3 development samples (see **Table A in S1 File**). Then, beginning with the 2 variables we identified as having the strongest empirical support from the literature (i.e., prior suicide attempts and prior psychiatric hospitalization), we evaluated if the combination (i.e., sum) of these 2 variables resulted in an AUC value that was consistently higher across the development samples than the AUC values for the individual variables when examined separately. Utilizing this general approach, we systematically evaluated each new variable for potential inclusion in the checklist until we were unable to identify any additional variables that improved discrimination of high-risk individuals from low-risk individuals in 1 or more of the development samples (see **Table 2**).

The final set of constructs selected for inclusion in the DRS are provided in **Table 2**, which also shows the impact of each variable’s entry into the model on the cumulative AUC value for different iterations of the DRS across the 3 development samples. It is, however, important to note that an iterative approach was taken to variable selection and that the constructs ultimately selected for inclusion in the DRS were those constructs that not only optimized predictive validity across the 3 development samples, but were also logical from both a theoretical and clinical perspective [18,29,30]. Other variables from the extant literature [3,18,21–23,29–43] were also considered (see **Table A in S1 File**), but not ultimately selected, including (among others) other psychiatric disorders (e.g., schizophrenia and anxiety disorders), recent life stressors, and various demographic variables (e.g., marital status). Different orders and iterations of variables (e.g., frequency, severity, and time frame of assessment) were also considered in order to optimize the predictive value of variables within the development samples. Please also note that many other potentially important variables (e.g., suicidal intent, access to lethal means, suicide plans, and a psychiatric hospitalization during the past 30 days) were not available for analysis in the samples utilized in the present study.

To be retained in the final version of the checklist, each variable needed to (1) have clear empirical support in the literature; (2) demonstrate a positive bivariate association with future suicide attempts in 1 or more of the development samples; (3) evidence incremental validity in 1 or more of the development samples; and (4) show minimal negative impact on incremental validity in the remaining development samples. Utilizing the approach described above, we initially selected 23 items for inclusion in the checklist, each weighted equally. Once we reached the point at which we were no longer able to identify any new variables that further improved the predictive utility of the score, we examined if doubling the weight by adding an additional point to the sum score of any of the items identified as top predictors further improved AUC values. This analysis revealed that doubling the weight of 4 of the top longitudinal predictors identified by Franklin and colleagues [3] (i.e., lifetime history of suicide attempts, psychiatric hospitalization, NSSI, and borderline personality disorder [BPD]) further improved the overall AUC value in 1 or more of the development samples (see **Table 2**).

### Evaluation of the Durham Risk Score

ROC curves and logistic regression analyses were used to evaluate the discriminative ability of the DRS across the samples. Signal detection analysis was used to identify an optimal cut score [52] and to develop risk groups to facilitate interpretation of scores. Concentration of risk was evaluated [23], and rates of attempts, risk ratios, ORs, and 95% CIs were calculated for risk groups. ROC curves were also calculated in subgroups of interest, including women, men, Black, White, Hispanic, lower-income individuals, younger adults, veterans, LGBTQ individuals, as well as individuals with and without a history of suicidal thoughts and behaviors.

### Missing data

Although we are strong proponents of multiple imputation and maximum likelihood estimation methods to handle missingness in most situations, we elected to treat missing data as absent (i.e., “0”) in the calculation of DRS scores in the present research because (1) this approach best reflects real-world clinical practice; and (2) some variables were systematically missing across different studies because they were not assessed as part of the study protocol. The only exception to this approach was for the VALOR sample, which was used to validate the DRS. Specifically, because the VALOR study protocol only assessed 15 of the 23 (i.e., 65%) variables used to calculate the DRS, VALOR analyses were limited to participating veterans (*N* = 780) with follow-up attempt data as well as complete data for all 15 of these variables to ensure that participants in the VALOR analyses had no more than 35% missing data.

### Measures

**Table B in S1 File** summarizes the items and measures used to assess each of the 23 constructs included in the DRS across studies. Measures used to index the various constructs included well-validated structured interviews, such as the C-SSRS [5], SITBI [49], the Structured Clinical Interview for DSM (SCID) [53], the Clinician-Administered PTSD Scale (CAPS) [54], the Mini International Neuropsychiatric Interview (MINI)] [55], and the Alcohol Use Disorder and Associated Disabilities Interview Schedule (AUDADIS) [56], as well as a variety of self-report instruments, including the Alcohol Use Disorders Identification Test (AUDIT) [57], Drug Abuse Screening Test (DAST) [58], Davidson Trauma Scale (DTS) [59], Patient Health Questionnaire-9 (PHQ-9) [60], Traumatic Life Events Questionnaire (TLEQ) [61], Childhood Trauma Questionnaire (CTQ) [62], Veterans Rand 12-Item Health Survey (VR-12) [63], the Life Events Checklist (LEC) [64], and the Symptom Checklist-90 (SCL-90) [65]. Study-specific questionnaires [25–27,44–47] were also used to assess constructs in some instances, particularly those related to demographic characteristics.

As can be seen in **Table B in S1 File**, in the vast majority of cases, all of the specific items used to assess the constructs included in the calculation of the DRS score were assessed at the time of the baseline assessment; however, there were 8 instances in which at least a part of a construct of interest was only assessed at the time of the Wave 2 assessment. Such items are clearly marked in bold in **Table B in S1 File**. In each case in which an item from a follow-up wave was included in the assessment of 1 of the 23 items included in the calculation of the DRS score, we carefully considered both the nature of the specific item as well as the nature of the construct in general before making an a priori decision about whether to use a specific variable from a specific sample in the calculation of the DRS. In each case where such a variable was included in the assessment of a given construct, we felt that inclusion of the data from a given item was justified, given that our overall goal was to make the best possible suicide attempt risk checklist in order to enhance clinical care.

Most of these instances occurred in the NESARC dataset. For example, childhood sexual abuse, childhood physical abuse, and being jailed or sent to a juvenile detention center prior to the age of 18 were not assessed at the time of the NESARC baseline assessment. We reasoned that, given that reporting of these items during adulthood would have still involved retrospective reporting, even if they had been administered during the baseline assessment, it was reasonable (though not ideal) to include information regarding these important childhood experiences from Wave 2 in calculating the DRS score. Information from the Wave 2 NESARC interview was also used to index PTSD, BPD, and NSSI. In the case of PTSD, interviewers were required to retrospectively establish if symptoms of PTSD had begun prior to the baseline interview and whether they had been present from the time of the baseline interview to the time of the time of the Wave 2 interview. In the case of BPD and NSSI (which was assessed as part of the BPD interview), because BPD is a personality disorder that should be present by early adulthood, NESARC interviewers were instructed to frequently precede BPD questions (including NSSI) with “Most of the time throughout your life, regardless of the situation or whom you were with…”. Given the way that these questions were asked, and the fact that BPD and NSSI are among the strongest predictors of suicide attempts [3,35], we felt that it was critical to include these items in the calculation of the DRS score. Sexual orientation was also not assessed at the time of the NESARC baseline assessment. Although we recognize that sexual orientation can change over time, given the importance of systematically assessing this construct in relation to risk for suicidal behavior [36], we felt that it was also important to include the sexual orientation variable collected at NESARC Wave 2 in the calculation of the DRS score.

We fully recognize the problems associated with including a subset of variables assessed cross-sectionally in a checklist designed to prospectively predict suicide attempts in clinical settings and would have strongly preferred to have only included variables assessed longitudinally in the present analyses; however, such an approach would have precluded us from including several of the most well-established longitudinal predictors of suicide attempts (e.g., NSSI) [3]. In recognition of this challenging situation, we conducted a sensitivity analysis in the NESARC dataset to evaluate the performance of the DRS when these variables were excluded from the calculation of the score. As subsequently described in the Results section below, we were pleased to find that this sensitivity analysis revealed that the DRS continued to perform quite well (AUC = 0.86) in the NESARC validation cohort when all variables collected at Wave 2 were excluded from the calculation of the score, indicating that the core DRS measure is, in fact, a robust prospective predictor of suicide attempts, regardless of whether the items assessed cross-sectionally are included or not.

Lifetime history of NSSI was also assessed cross-sectionally in the VALOR sample. Again, however, the interviewers for this study were required to determine if the participant’s history of NSSI was present prior to the baseline interview, and only individuals who were retrospectively determined to have engaged in NSSI prior to the baseline interview were coded as having a history of lifetime NSSI at the time of the baseline interview. Given the manner in which NSSI was assessed in VALOR, as well as the importance of NSSI to suicide attempt risk prediction [3], we felt that inclusion of this data was also justified, given our primary goal of developing the best possible clinical assessment to facilitate identification of at-risk individuals.

## Results

### Descriptive statistics and distribution of scores

Descriptive statistics for the DRS (**Fig 1**) across the different samples are provided in **Table 1**. As would be expected for a suicide attempt risk score, the DRS was positively skewed (1.7) and kurtotic (4.7) in the overall sample (**Fig B in S1 File**); however, among the 288 individuals who made a prospective suicide attempt during the follow-up period, DRS scores were normally distributed (*M* = 9.9; *SD* = 4.4; skewness = 0.33; kurtosis = −0.7; range: 1 to 22; **Fig C in S1 File**).

### Logistic regression analyses

Logistic regression analyses were conducted to examine the predictive utility of the continuous DRS score as a predictor of prospective suicide attempts across the samples (**Table 3**). These analyses revealed a consistent pattern of increasing risk as a function of DRS score in both the combined development (*OR* = 1.48, 1.43 to 1.53, *p* < 0.001; Nagelkerke pseudo *R*^2^ = 0.27) and validation samples (*OR* = 1.51, 1.46 to 1.56, *p* < 0.001; Nagelkerke pseudo *R*^2^ = 0.29). Thus, for each additional point increase on the DRS, the odds of making a prospective suicide attempt increased by approximately 50% in both the combined development and validation cohorts.

**Table 3:** Summary of logistic regression findings.

**Logistic regression results using the DRS to predict suicide attempts across the development and validation samples**												
**Sample**		***N***	**# Attempted**	**Intercept**	**DRS beta**	**SE**	**Nagelkerke pseudo R**^**2**^			**OR**	**OR 95% CI**	***p***
NESARC 1		8,872	62	−7.52	0.43	0.03	0.33			**1.54**	1.46–1.63	<0.001
NESARC 2		8,525	57	−6.87	0.35	0.03	0.21			**1.42**	1.35–1.50	<0.001
REHAB		233	10	−5.86	0.48	0.12	0.29			**1.62**	1.29–2.03	<0.001
Combined development cohort		17,630	129	−7.09	0.39	0.02	0.27			**1.48**	1.43–1.53	<0.001
NESARC 3		8,516	62	−7.29	0.41	0.03	0.29			**1.51**	1.43–1.59	<0.001
NESARC 4		8,728	60	−7.43	0.42	0.03	0.31			**1.52**	1.44–1.61	<0.001
VALOR		780	37	−5.82	0.38	0.06	0.18			**1.46**	1.30–1.65	<0.001
Combined validation cohort		18,024	159	−7.15	0.41	0.02	0.29			**1.51**	1.46–1.56	<0.001
**Rates of prospective suicide attempts, RRs, and ORs by risk group status in the combined development sample (*N* = 17,630)**												
**Suicide risk group**	**Range**	**% of sample**	**Cumulative %**	**Controls**	**Attempters**	**Total**	**Rate**	**RR**	**Odds**	**OR**	**OR 95% CI**	***p***
Lowest risk	0–2	45.7%	45.7%	8,055	2	8,057	0.02%	1.0	0.0002	**1.0**		
Low risk	3–5	40.4%	86.1%	7,095	24	7,119	0.3%	13.6	0.003	**13.6**	3.2–57.7	<0.001
Moderate risk	6–8	9.4%	95.5%	1,630	34	1,664	2.0%	82.3	0.02	**84.0**	20.2–350.0	<0.001
High risk	9–11	3.0%	98.6%	507	28	535	5.2%	210.8	0.06	**222.4**	252.8–936.3	<0.001
Very high risk	12–14	0.9%	99.5%	141	21	162	13.0%	522.2	0.15	**599.8**	139.3–2,582.7	<0.001
Highest risk	15–30	0.5%	100.0%	73	20	93	21.5%	866.3	0.27	**1,103.4**	253.3–4,807.2	<0.001
Total				17,501	129	17,630	0.7%					
**Rates of prospective suicide attempts, RRs, and ORs by risk group status in the combined validation sample (*N* = 18,024)**												
**Suicide risk group**	**Range**	**% of sample**	**Cumulative %**	**Controls**	**Attempters**	**Total**	**Rate**	**RR**	**Odds**	**OR**	**OR 95% CI**	***p***
Lowest risk	0–2	43.5%	43.5%	7,844	2	7,846	0.03%	1.0	0.0003	**1.0**		
Low risk	3–5	40.8%	84.3%	7,332	24	7,356	0.3%	12.8	0.003	**12.8**	3.0–54.3	0.001
Moderate risk	6–8	10.4%	94.8%	1,845	36	1,881	1.9%	75.1	0.02	**76.5**	18.4–318.1	<0.001
High risk	9–11	3.6%	98.3%	600	41	641	6.4%	250.9	0.07	**268.0**	64.7–1,110.7	<0.001
Very high risk	12–14	1.1%	99.4%	176	24	200	12.0%	470.8	0.14	**534.8**	125.4–2,280.5	<0.001
Highest risk	15–30	0.6%	100.0%	68	32	100	32.0%	1255.4	0.47	**1,845.6**	433.6–7,855.3	<0.001
Total				17,865	159	18,024	0.9%					
**Rates of prospective suicide attempts, RRs, and ORs when using a cut score of ≥6**												
	**DRS <6**				**DRS ≥6**				**RR**	**OR**	**OR 95% CI**	***p***
	**Controls**	**Attempts**	**Rate**	**Odds**	**Controls**	**Attempts**	**Rate**	**Odds**				
Combined development	15,150	26	0.2%	0.002	2,351	103	4.2%	0.04	24.5	**25.5**	16.6–39.3	<0.001
Combined validation	15,176	26	0.2%	0.002	2,689	133	4.7%	0.05	27.6	**28.9**	18.9–44.0	<0.001

CI, confidence interval; DRS, Durham Risk Score; NESARC, National Epidemiologic Survey on Alcohol and Related Conditions; OR, odds ratio; REHAB, Assessing and Reducing Post-Deployment Violence Risk; RR, risk ratio; SE, standard error; VALOR, Veterans After-Discharge Longitudinal Registry.

### Receiver operating characteristic curve analyses

**Fig 2:**
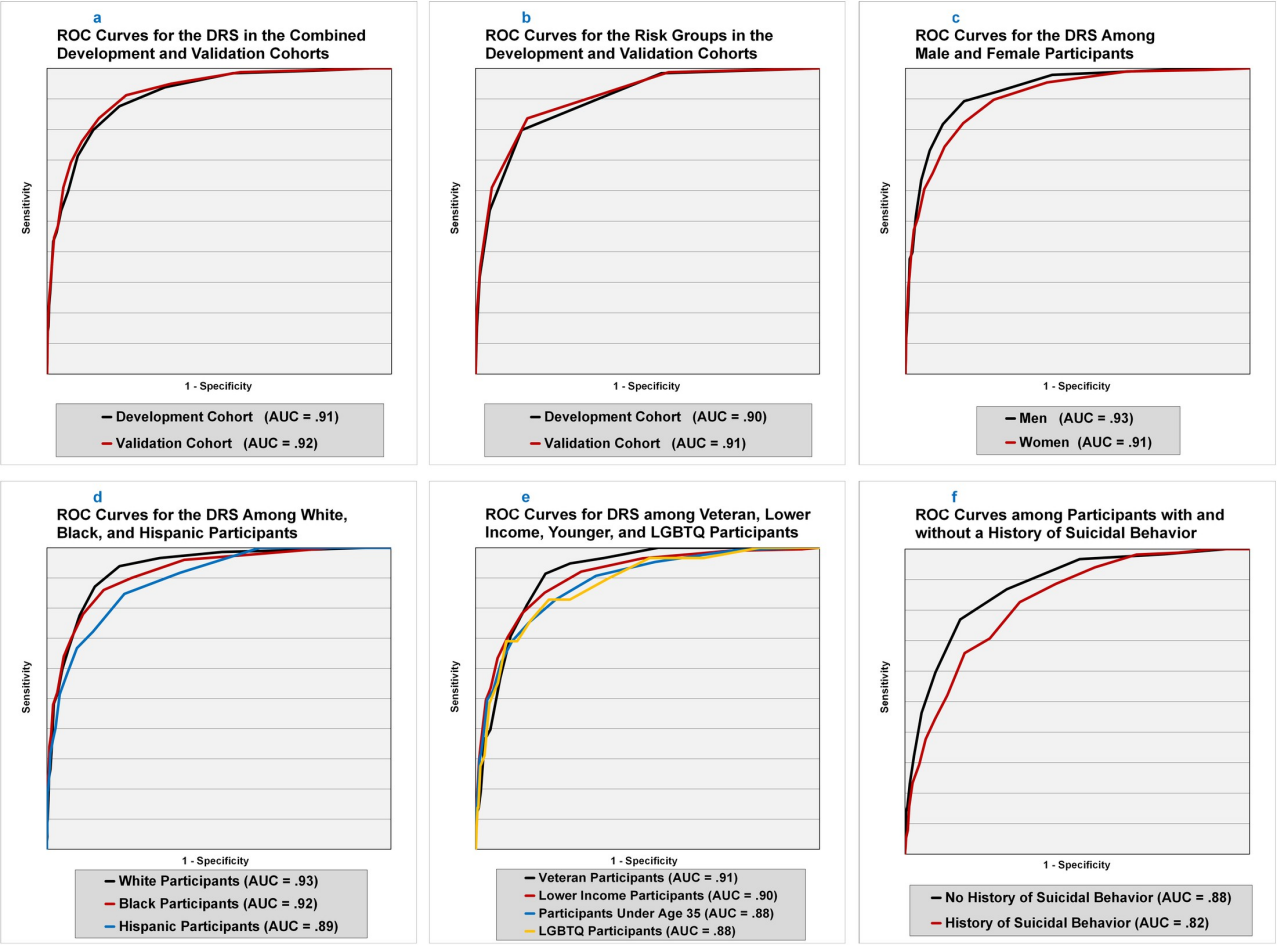
ROC curves for the DRS across subsets of participants. Fig 2a: ROC curves for the DRS in the combined development and validation cohorts. Black line = development cohort; red line = validation cohort. Fig 2b: ROC curves for the risk groups in the development and validation cohorts. Black line = development cohort; red line = validation cohort. Fig 2c: ROC curves for the DRS among male and female participants. Black line = men; red line = women. Fig 2d: ROC curves for the DRS among White, Black, and Hispanic participants. Black line = White participants; red line = Black participants; blue line = Hispanic participants. Fig 2e: ROC curves for DRS among veteran, lower-income, younger, and LGBTQ participants. Black line = veteran participants; red line = lower-income participants; blue line = participants under the age of 35; orange line = LGBTQ participants. AUC, area under the curve; DRS, Durham Risk Score; LGBTQ, lesbian, gay, bisexual, transgender, and queer or questioning; ROC, receiver operating characteristic.

ROC analyses revealed that the overall AUC for the DRS total score in the combined development sample (total *N* = 17,630; **Table 4**) was 0.91 (0.89 to 0.93; **Fig 2A**). More importantly, the DRS continued to demonstrate excellent discriminative ability in the 3 validation samples excluded from the development analyses (combined validation sample AUC = 0.92, 0.90 to 0.94, *N* = 18,024; **Table 4**, **Fig 2A**), suggesting that our approach to instrument development was successful in protecting against overfitting [50].

**Table 4:** Areas under the curve for DRS and risk group status across samples and subsets of participants.

		DRS	DRS	DRS	Risk group	Risk group	Risk group
Sample/subset of participants	*N*	AUC	AUC SE	AUC 95% CI	AUC	AUC SE	AUC 95% CI
NESARC 1	8,872	0.93	0.02	0.90–0.96	0.91	0.02	0.87–0.95
NESARC 2	8,525	0.89	0.02	0.85–0.93	0.88	0.02	0.83–0.92
REHAB	233	0.88	0.05	0.78–0.98	0.89	0.04	0.81–0.97
Combined development sample	**17,630**	**0.91**	**0.01**	**0.89**–**0.93**	**0.90**	**0.01**	**0.87**–**0.92**
NESARC 3	8,516	0.92	0.02	0.88–0.95	0.90	0.02	0.86–0.94
NESARC 4	8,728	0.92	0.02	0.88–0.95	0.90	0.02	0.86–0.94
VALOR	780	0.82	0.03	0.77–0.87	0.80	0.03	0.74–0.86
Combined validation sample	**18,024**	**0.92**	**0.01**	**0.90**–**0.94**	**0.91**	**0.01**	**0.88**–**0.93**
Female participants	20,523	0.91	0.01	0.89–0.93	0.89	0.01	0.87–0.91
Male participants	15,130	0.93	0.01	0.90–0.95	0.92	0.01	0.89–0.94
Black participants	6,814	0.92	0.02	0.88–0.96	0.90	0.02	0.86–0.95
White participants	20,886	0.93	0.01	0.91–0.95	0.92	0.01	0.90–0.94
Hispanic participants	6,458	0.89	0.02	0.86–0.93	0.87	0.02	0.83–0.91
Veteran participants	2,859	0.91	0.01	0.89–0.94	0.90	0.01	0.88–0.93
Lower-income participants	19,167	0.90	0.01	0.88–0.92	0.88	0.01	0.86–0.91
Participants under 35 years of age	10,711	0.88	0.02	0.85–0.91	0.87	0.02	0.83–0.90
LGBTQ participants	747	0.88	0.03	0.81–0.94	0.86	0.04	0.79–0.93
Participants with a history of suicidal behavior at baseline	3,489	0.82	0.02	0.79–0.85	0.81	0.02	0.78–0.84
Participants without a history of suicidal behavior at baseline	32,165	0.88	0.02	0.85–0.91	0.85	0.02	0.81–0.88

AUC, area under the curve; CI, confidence interval; DRS, Durham Risk Score; LGBTQ, lesbian, gay, bisexual, transgender, and queer or questioning; NESARC, National Epidemiologic Survey on Alcohol and Related Conditions; REHAB, Assessing and Reducing Post-Deployment Violence Risk; SE, standard error; VALOR, Veterans After-Discharge Longitudinal Registry.

### Subgroup analyses

Subgroup-based ROC analyses (**Table 4**, **Fig 2C–2E**) revealed that the DRS performed well among women (AUC = 0.91, 0.89 to 0.93), men (AUC = 0.93, 0.90 to 0.95), Black (AUC = 0.92, 0.88 to 0.96), White (AUC = 0.93, 0.91 to 0.95), Hispanic (AUC = 0.89, 0.86 to 0.93), veterans (AUC = 0.91, 0.89 to 0.94), lower-income individuals (AUC = 0.90, 0.88 to 0.92), younger adults (AUC = 0.88, 0.85 to 0.91), and LGBTQ individuals (AUC = 0.88, 0.81 to 0.94). In addition, as expected, participants with a history of suicidal thoughts or behaviors (*N* = 3,489) were significantly more likely to make a prospective suicide attempt (4.8% versus 0.4%; *OR* = 13.3, 10.5 to 16.9, *p* < 0.001) than those without a history of suicidal thoughts or behaviors at baseline; however, even within this high-risk subgroup, the DRS demonstrated good utility (AUC = 0.82, 0.79 to 0.85; **Fig 2F**). We further observed that 42% of prospective suicide attempts occurred among individuals who reported no lifetime history of suicidal thoughts or behavior at the time of the baseline assessment. Notably, the DRS (AUC = 0.88, 0.85 to 0.91; **Fig 2F**) also performed well in this important, but understudied subgroup.

### Concentration of risk

**Fig 3:**
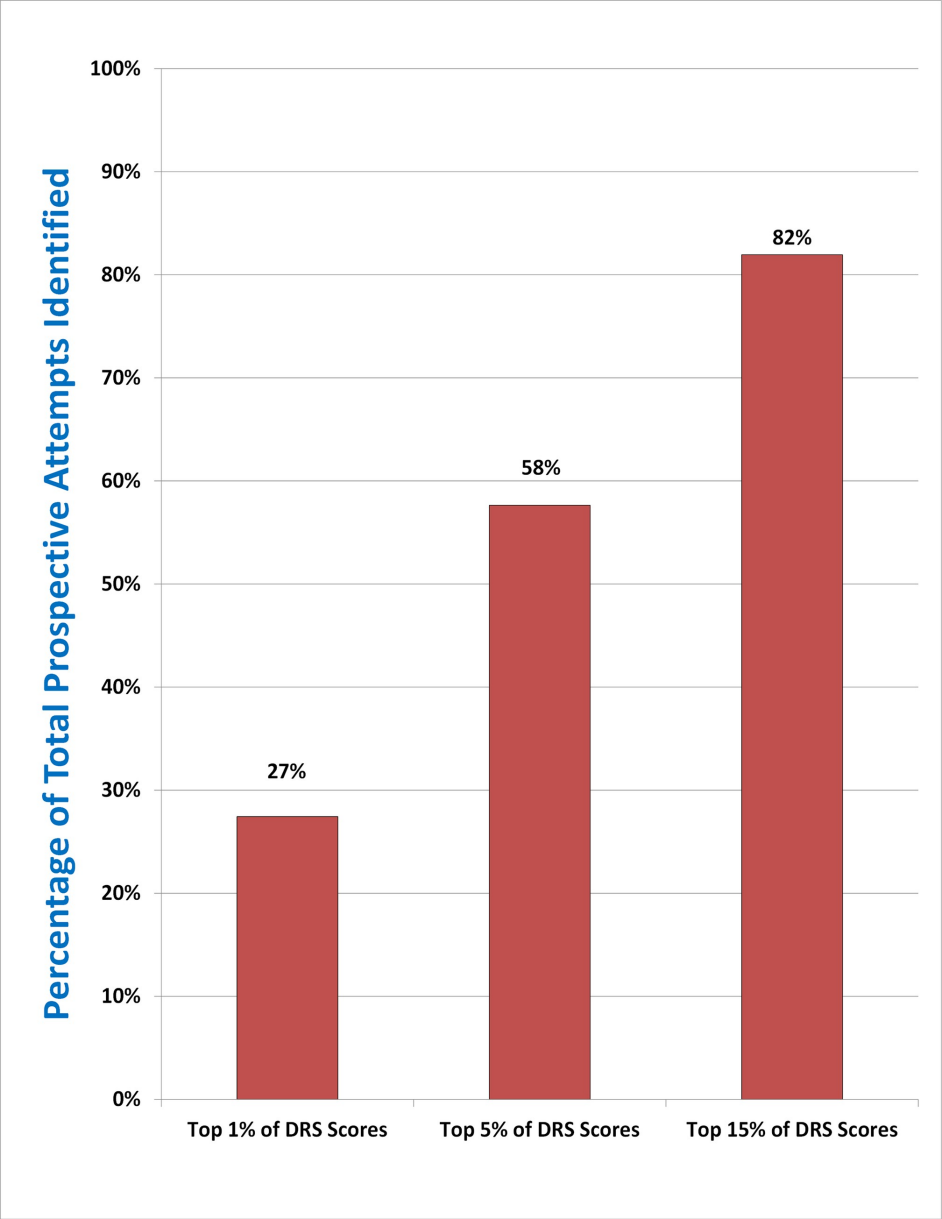
Concentration of risk of prospective suicide attempts across all participants (*N* = 35,654). DRS, Durham Risk Score.

A concentration of risk analysis [23] found that across all 35,654 participants, 82% of observed prospective suicide attempts occurred among individuals in the top 15% of DRS scores; 58% occurred in the top 5%; and 27% occurred in the top 1% (**Fig 3**).

### Signal detection analyses

Signal detection analysis was used to identify a cut score that simultaneously maximized sensitivity and specificity and would be appropriate in typical clinical screening situations (**Table 5**). For situations in which a single clinical cut score is needed to identify at-risk individuals, we recommend a cut score of 6 or greater (corresponding to moderate-risk group status or approximately top 15% of scores), as this score had the highest overall J statistic [52] (i.e., Youden index = 0.68) and maximized both sensitivity (82%) and specificity (86%). A cut score of 6 also produced a PPV of 4% and a negative predictive value (NPV) of 100%. In contrast, for situations in which higher levels of PPV are preferred, a cut score of 9 or higher (corresponding to high-risk group status or approximately top 5% of scores) resulted in specificity of 96%, sensitivity of 58%, PPV of 10%, and NPV of 100%, whereas a cut score of 15 or higher (corresponding to highest-risk group status or approximately top 0.5% of scores) resulted in specificity of 100%, sensitivity of 18%, PPV of 27%, and NPV of 99% (**Table 5**).

**Table 5:** Summary of the signal detection analyses for different DRS cutoffs in the total sample (*N* = 35,654).

Risk group	Cutoff	Youden index (J)	Sensitivity	Quality of sensitivity k(1)	Specificity	Quality of specificity k(0)	Efficiency	Quality of efficiency k(0.5)	PPV	NPV	AUC	AUC 95% CI	OR	OR 95% CI	*p*
**Lowest-risk group**	≥1	0.06	1.00	1.00	0.06	0.00	0.07	0.00	0.01	1.00	0.53	0.50–0.56	^a^	^a^	
	≥2	0.22	0.99	0.97	0.22	0.00	0.23	0.00	0.01	1.00	0.61	0.58–0.64	41.5	10.3–166.8	<0.001
**Low-risk group**	≥3	0.44	0.99	0.97	0.45	0.01	0.45	0.01	0.01	1.00	0.72	0.70–0.74	58.0	21.6–155.6	<0.001
	≥4	0.59	0.94	0.91	0.65	0.01	0.65	0.03	0.02	1.00	0.80	0.78–0.82	31.4	19.0–52.0	<0.001
	≥5	0.68	0.90	0.87	0.78	0.02	0.78	0.05	0.03	1.00	0.84	0.82–0.86	30.5	20.9–44.6	<0.001
**Moderate-risk group**	≥6	0.68	0.82	0.79	0.86	0.04	0.86	0.07	0.04	1.00	0.84	0.82–0.86	27.3	20.2–36.9	<0.001
	≥7	0.64	0.74	0.71	0.90	0.05	0.90	0.10	0.06	1.00	0.82	0.79–0.85	27.0	20.7–35.2	<0.001
	≥8	0.58	0.65	0.62	0.94	0.07	0.93	0.12	0.08	1.00	0.79	0.76–0.83	26.7	20.9–34.1	<0.001
**High-risk group**	≥9	0.53	0.58	0.55	0.96	0.09	0.95	0.15	0.10	1.00	0.77	0.73–0.80	29.4	23.1–37.3	<0.001
	≥10	0.45	0.48	0.46	0.97	0.11	0.97	0.18	0.12	1.00	0.72	0.69–0.76	29.7	23.4–37.7	<0.001
	≥11	0.42	0.44	0.43	0.98	0.15	0.98	0.22	0.16	1.00	0.71	0.67–0.75	40.0	31.3–51.1	<0.001
**Very high–risk group**	≥12	0.32	0.34	0.33	0.99	0.17	0.98	0.22	0.17	0.99	0.66	0.62–0.70	38.7	29.8–50.3	<0.001
	≥13	0.27	0.27	0.27	0.99	0.19	0.99	0.22	0.20	0.99	0.63	0.59–0.67	42.1	31.7–55.7	<0.001
	≥14	0.23	0.24	0.23	0.99	0.24	0.99	0.23	0.24	0.99	0.62	0.58–0.65	51.3	37.8–69.4	<0.001
**Highest-risk group**	≥15	0.18	0.18	0.18	1.00	0.26	0.99	0.21	0.27	0.99	0.59	0.55–0.63	55.0	39.1–77.6	<0.001
	≥16	0.14	0.14	0.14	1.00	0.35	0.99	0.20	0.35	0.99	0.57	0.53–0.61	76.9	51.3–115.3	<0.001
	≥17	0.09	0.09	0.09	1.00	0.38	0.99	0.14	0.39	0.99	0.55	0.51–0.58	85.5	51.5–141.8	<0.001
	≥18	0.04	0.05	0.04	1.00	0.33	0.99	0.08	0.33	0.99	0.52	0.49–0.56	64.3	32.7–126.4	<0.001
	≥19	0.03	0.03	0.03	1.00	0.45	0.99	0.06	0.45	0.99	0.52	0.48–0.55	103.7	42.6–252.2	<0.001
	≥20	0.01	0.01	0.01	1.00	0.60	0.99	0.02	0.60	0.99	0.51	0.47–0.54	186.1	31.0–1,118.1	<0.001

^a^An OR could not be calculated for a cutoff score of ≥1 because no prospective suicide attempts occurred among the 2,113 participants who had a DRS score of 0.

AUC, area under the curve; CI, confidence interval; DRS, Durham Risk Score; NPV, negative predictive value; OR, odds ratio; PPV, positive predictive value.

### Suicide attempt risk groups

**Fig 4:**
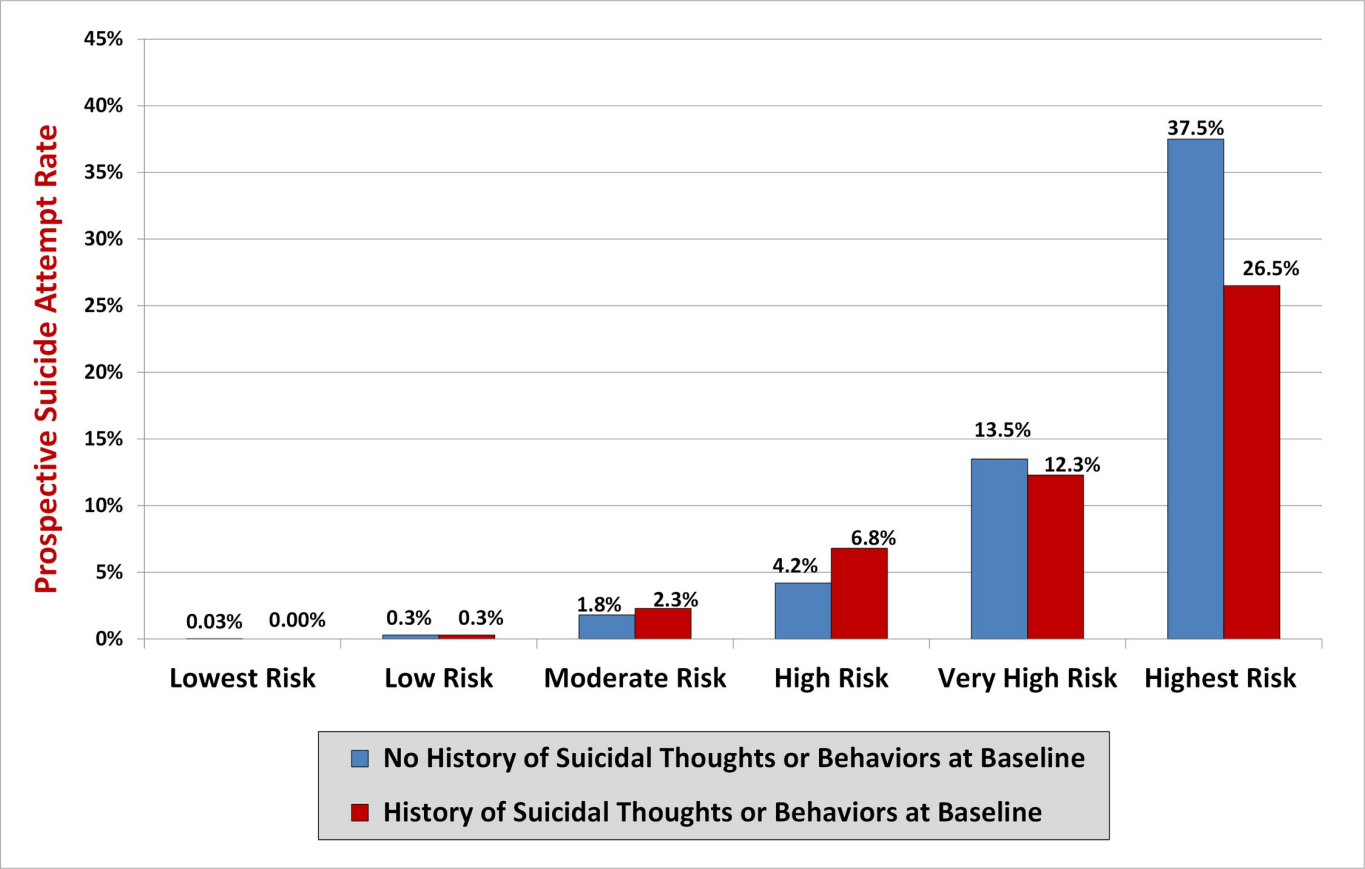
Rates of attempts by risk group status among participants with and without a history of suicidal thoughts and behaviors (*N* = 35,654).

To facilitate rapid interpretation of scores, suicide attempt risk groups were identified that appeared to correspond to clinically meaningful increases in suicide attempt risk based on the signal detection analyses described above. **Table C in S1 File** provides rates of attempts, odds, and predicted probabilities by risk group status for the total sample (*N* = 35,654). The AUC for risk group status was 0.90 (0.87 to 0.92) in the development sample and 0.91 (0.88 to 0.93) in the validation sample (**Table 4**, **Fig 2B**), indicating that our 6-group classification system was nearly as accurate at predicting future attempts as the DRS total score. Moreover, as expected, each increasing group level was associated with a marked increase in risk (**Table 3**). For example, the odds of making a prospective suicide attempt were more than 1,800 times greater in the highest-risk group (top 0.6% of scores; suicide attempt rate = 32.0%) relative to the lowest-risk group (bottom 43.5% of scores; suicide attempt rate = 0.03%) in the validation cohort (*OR* = 1,845.6, 433.6 to 7,855.3, *p* < 0.001; **Table 3**). Notably, as was the case for the total DRS score, risk group status appeared to confer similar increases in risk to participants regardless of whether or not they reported a history of suicidal thoughts or behavior at the time of the baseline assessment (**Table 4**, **Fig 4**).

### Sensitivity analysis to examine the impact of variables assessed at Wave 2

To assess the potential impact of the variables that were assessed at Wave 2 in the NESARC study on the performance of the DRS, a sensitivity analysis was conducted in which the 7 items from Wave 2 (i.e., sexual abuse/assault, child physical abuse, history of juvenile detention, NSSI, BPD, PTSD, and LGBTQ status) were excluded from the computation of the DRS score. Please note that item #W2S11Q6A (which was also assessed at Wave 2) was also removed from the calculation of lifetime history of violence and incarceration for these analyses. Thus, all variables used in the calculation of the DRS in these sensivity analyses were assessed at the time of the Wave 1 interview and prior to the occurence of any prospective suicide attempts that occurred between Waves 1 and 2. This analysis (**Table D in S1 File**) revealed that the abbreviated version of the DRS based entirely on variables collected during the baseline assessment continued to perform quite well (AUC = 0.86) in the combined NESARC validation cohort (i.e., NESARC 3 and NESARC 4, combined *N* = 17,244).

### Association between number of items assessed and AUC values

A robust positive correlation was observed between number of items assessed and AUC values across the 6 samples (*r* = 0.94, *p* = 0.006; see **Fig D1 in S1 File**). Accordingly, we recommend that, whenever possible, clinicians and researchers who wish to use the DRS systematically assess and score all 23 items using the most reliable and valid assessment methods available to them at the time of the assessment (see **S1 Durham Risk Score Guide** for additional details). We also assessed the association between number of items and AUC values with the 4 additional sensitivity samples included (**Fig D2 in S1 File**) and observed that there continued to be a robust positive association between number of items assessed and AUC values (*r* = 0.83, *p* = 0.003). Moreover, as can be seen in **Table D** and **Fig D2 in S1 File**, 3 of the 4 NESARC sensitivity samples (AUC range: 0.85 to 0.87) had AUC values that were higher than the AUC for the VALOR sample (0.82), which also only assessed 15 items. Thus, the lower AUC values observed in the NESARC sensitivity analyses (i.e., when only 15 items were included in the calculation of the total score) were highly consistent with the pattern of findings one would expect based on the positive association observed between items assessed and AUC values.

### Prediction of suicide attempts occurring outside of the context of mood disorders

During the peer review process, reviewers noted that a potential limitation of our choice to utilize the question from the Low Mood portion of the NESARC interview to define prospective suicide attempts (i.e., “During that time since your LAST interview when (your mood was at its lowest/you enjoyed or cared least about things), did you attempt suicide?”) was that our findings might be not be generalizable to the prediction of suicide attempts occurring outside of the context of mood disorders (e.g., during psychosis). To address this important potential limitation, we conducted an additional sensitivity analysis that utilized a set of questions from the NESARC Wave 2 assessment that were not utilized in the development of the DRS. Specifically, during a different portion of the NESARC Wave 2 interview, participants were also asked, “In your entire life, did you ever attempt suicide?” If participants answered affirmatively, they were then also asked “How old were you the first time?” and “How old were you the most recent time?” [22]. While some researchers interested in developing predictive models of suicide attempts within NESARC have defined their primary outcome as the most recent attempt having occurred within 3 years of participants’ age at Wave 2 [22], we instead elected to utilize the question from the Low Mood portion of the Wave 2 interview to develop the DRS because (1) it specifically inquired about new instances of suicide attempts occurring since the Wave 1 interview; (2) the number of days between Wave 1 and Wave 2 was variable, making it impossible to definitively determine if a suicide attempt occurring within 3 years of participants’ age at the time of the Wave 2 interview actually occurred after the Wave 1 assessment; and (3) the suicide attempt question from the Low Mood portion of the interview actually identified a greater number of prospective suicide attempts (241 versus 222).

To assess the potential impact of our choice to use the suicide attempt question from the Low Mood portion of the NESARC as our primary outcome variable, we calculated ROC curves for the DRS in the combined NESARC validation cohort (i.e., NESARC 3 and NESARC 4, combined *N* = 17,244) utilizing 3 different outcomes: (a) suicide attempts occurring since the last interview based on the Low Mood portion of the Wave 2 interview (i.e., our original operational definition which we used to develop the DRS), which resulted in 122 suicide attempt cases and 17,122 controls in the combined NESARC validation cohort; AUC = 0.92, 0.89 to 0.94; (b) most recent suicide attempt having occurred within 3 years of participants’ age at Wave 2 [22], which resulted in 121 suicide attempt cases and 17,123 controls, AUC = 0.91, 0.88 to 0.93; and (c) suicide attempts identified by either method, which resulted in 175 suicide attempt cases and 17,069 controls, AUC = 0.91, 0.88 to 0.93. Thus, the AUC values for the DRS across these 3 different suicide attempt definitions were remarkably similar, ranging from 0.91 to 0.92, and had highly overlapping 95% CIs. It should also be noted that suicide attempts were not assessed within the context of mood disorders in either REHAB or VALOR. Thus, our initial findings suggest that the DRS is similarly effective at identifying risk for future suicide attempts that occur outside of the context of mood disorders.

### Comparison of the DRS with a logistic regression–derived risk score

During the peer review process, while noting the attractiveness of the simplistic scoring approach we utilized because of the ease with which it could be manually calculated by clinicians, reviewers requested that we also explore whether more specific weights derived directly from a logistic regression model might further improve the predictive performance of the DRS within the NESARC sample. Accordingly, we conducted an additional logistic regression on the 23 variables used to calculate the DRS in the combined NESARC development cohort (i.e., NESARC 1 and NESARC 2, combined *N* = 17,397). As can be seen in **Table E in S1 File**, the variables most strongly associated with prospective suicide attempts in the logistic regression model included BPD (*AOR* = 5.87, 3.65 to 9.43, *p* < 0.001), lifetime NSSI (*AOR* = 3.52, 2.12 to 5.85, *p* < 0.001), LGBTQ status (*AOR* = 3.18, 1.66 to 6.11, *p* = 0.001), lifetime psychiatric hospitalization (*AOR* = 2.58, 1.47 to 4.50, *p* = 0.001), and poor perceived health (*AOR* = 2.07, 1.32 to 3.25, *p* = 0.001). In contrast, the variables with the weakest association with prospective suicide attempts in the logistic regression model included psychiatric hospitalization in the past year, lifetime mood disorder, weekly binge drinking, lower income, and having less than a high school education (all *p*’s > 0.45). Next, we used the regression coefficients from the logistic regression model conducted in the NESARC development cohort as risk score weights for the 23 variables. We then calculated ROC curves to compare the predictive validity of the DRS with the logistic regression–derived risk score in the NESARC validation cohort (i.e., NESARC 3 and NESARC 4, combined *N* = 17,244), which included 122 suicide attempt cases and 17,122 controls. This analysis revealed that the DRS (AUC = 0.92, 0.89 to 0.94) performed quite similarly to the logistic regression–derived score (AUC = 0.91, 0.88 to 0.94), despite using a much simpler scoring approach. In addition, DeLong test confirmed that the AUCs for the 2 models were not significantly different (*z* = 0.66, *p* = 0.51), providing additional support for our overall approach to measurement development, which maximized predictive utility while still providing clinicians with a simple scoring approach that can be calculated by hand.

### Comparison with the SAD PERSONS scale

During the peer review process, reviewers also requested that we directly compare the DRS with the SPS [12] within the same dataset. The SPS is an acronym and mnemonic device developed by Patterson and colleagues in 1983 [12] to guide assessment of suicide risk. The scale is widely utilized [11] and was specifically developed to teach medical students how to assess suicide risk [12]. Patients are assigned 1 point for each of the 10 risk factors that are deemed to be present by the clinician at the time of the assessment. The specific risk factors to be assessed include: **S**ex, **A**ge, **D**epression, **P**revious attempt, **E**thanol abuse, **R**ational thinking loss, **S**ocial supports lacking, **O**rganized plan, **N**o spouse, and **S**ickness [12]. We developed scoring for the SPS in the NESARC sample since this study included reasonable assessments for 9 of the 10 SPS items (see **Table F in S1 File** for details on scoring procedures for the SPS in NESARC). The only item that was not directly assessed in NESARC was “Organized plan,” for which we substituted lifetime suicidal ideation, which, notably, had the second highest overall bivariate AUC across the development samples (average AUC = 0.72; see **Table A in S1 File**). The SPS (*M* = 2.8; *SD* = 1.6; range: 0 to 10) exhibited an AUC of 0.74 (0.69 to 0.79) in the combined NESARC validation cohort (combined *N* = 17,244), which was a better performance than it has shown in some prior studies [13]; however, this value was only slightly better than the AUC for lifetime suicidal ideation by itself in the NESARC validation cohort (AUC = 0.72, 0.66 to 0.77) and was significantly worse than the AUC for the DRS (AUC = 0.92, *z* = 8.2, *p* < 0.0001), indicating that the DRS was significantly better than the SPS at predicting future suicide attempts in the NESARC validation cohort.

## Discussion

Taken together, the findings from the present research suggest that the DRS is a promising new tool that has the potential to enhance clinicians’ ability to identify individuals at risk for attempting suicide in the future. As described above, recent studies indicate that neither clinical judgment [10,19]nor existing suicide risk assessments are sufficiently accurate at predicting future suicide attempts [3,4,10,13,14,17]. Belsher and colleagues [24] have further noted that current risk models have a poor balance between sensitivity and specificity. Thus, the fact that our recommended cut score of 6 for typical screening situations (corresponding to moderate-risk group status or higher) produced a sensitivity value of 82% and a specificity value of 86% is highly encouraging, as we believe that these values represent a reasonable balance between sensitivity and specificity for a suicide attempt risk screen.

Importantly, these values also exceed the guidelines set forth by Runeson and colleagues [17] as sufficient to guide clinical decision-making. They also exceed the threshold accuracy values identified by Ross and colleagues [66] as necessary for suicide risk prediction to be combined with an active contact and follow-up intervention to become cost-effective from a healthcare sector perspective. Additionally, a cut score of 9 or greater on the DRS (corresponding to high-risk group status or approximately top 5% of scores) produces specificity (96%), sensitivity (58%), and PPV (10%) values that exceed the cost-effectiveness threshold accuracy values identified by Ross and colleagues [66] as necessary for suicide risk prediction to be combined with more intensive (and expensive) cognitive behavioral therapy interventions. Thus, while more work is needed to prospectively evaluate the utility of the DRS in actual healthcare settings, our initial findings suggest that the DRS has the potential to significantly enhance clinical care for patients in a cost-effective manner.

Another common criticism of existing suicide risk assessment methods is that they fail to provide clinicians with probability scores to guide decision-making [24]. Moreover, whereas some existing clinical assessments (e.g., SPS [12]) provide clinical guidelines for different risk scores, the relatively poor performance of these scales suggests that such guidance may be unfounded and inappropriate [13]. Thus, an additional strength of the DRS is that it provides clinicians with a means of efficiently classifying patients’ risk for attempting suicide into 1 of 6 different risk groups, with each subsequent risk group corresponding to an increasing probability of attempting suicide in the future. Importantly, risk group status was highly predictive of suicide attempts (AUC = 0.91) in the combined validation cohort, suggesting that these risk groups do, in fact, correspond to clinically meaningful increases in risk. Further evidence for the clinical utility of these risk groups comes from the fact that individuals in the lowest-risk (44.6% of the total sample) and low-risk (40.6% of the total sample) groups had rates of suicide attempts (0.03% and 0.3%, respectively) that were well below the national average in the US, which is presently 0.6% annually [67]. In contrast, the rates of attempts observed in the moderate-risk (2%), high-risk (6%), very high–risk (12%), and highest-risk (27%; **Table C in S1 File**) groups were all substantially higher than the annual rate in the US. Indeed, within the validation cohort, we observed that the odds of making a prospective suicide attempt were more than 1,800 times greater in the highest-risk group relative to the lowest-risk group (*OR* = 1,845.6; *p* < 0.001; **Table 3**), providing strong support for the clinical utility of this 6-group classification system. The fact that these 6 suicide attempt risk categories can be quickly and efficiently derived from raw scores is another particularly noteworthy strength of the DRS.

To further contextualize the present findings, it is also noteworthy that the lowest AUC value for the DRS observed across all samples and subgroups examined (0.82; **Table 4**) was equivalent to the largest C-statistic (0.82; AUC equivalent) reported for all external validations of the Framingham Risk Score in a recent meta-analysis (range: 0.55 to 0.82) [28]. In contrast, as noted above, multiple studies suggest that AUC values for existing suicide attempt risk assessments (including the recently developed Oxford Mental Illness and Suicide tool [68]) generally fall near or below 0.72 [3,4,10,13,68]. Moreover, a direct comparison of the DRS with the SPS [12]—one of the most commonly used suicide risk assessments in the world [11]—confirmed that the DRS significantly outperformed this widely used suicide risk algorithm in the combined NESARC validation cohort (AUC: 0.92 versus 0.74; *z* = 8.2, *p* < 0.0001).

While no conclusions can be drawn in the absence of direct comparisons with other existing suicide attempt risk assessment models, our initial findings suggest that the diagnostic accuracy of the DRS is likely to be higher than that of many existing suicide attempt risk assessment models [3,4,10–13,68], and similar to, or better than, other widely implemented clinical algorithms [11,12,28]. It is also notable that the AUC for the DRS in the combined validation cohort was similar to the cross-validated AUC of a recently published machine learning suicide attempt risk algorithm developed from the same population of NESARC participants that was derived from nearly 3,000 different baseline features [22]. Thus, our initial findings suggest that the diagnostic accuracy of the DRS may also be similar to that of a machine learning–based model developed within the same population [22], despite the fact that it contains far fewer items and can be manually calculated by a clinician.

### Study limitations and future directions

It should, however, also be emphasized that the present findings are based entirely on secondary analyses of rigorously collected, prospective research data. As a result, the degree to which the current findings will generalize to other settings, including clinical settings, is unknown at the present time. Thus, additional prospective research is still needed to validate the DRS in independent samples and to determine how best to assess each of the constructs used to calculate it. Relatedly, it is also important to note that there is presently no empirical support for the DRS in situations in which clinicians rely exclusively on their clinical impressions of patients to calculate the DRS score (as opposed to using standardized instruments to assess each of the 23 DRS constructs). Accordingly, we strongly recommend that clinicians who wish to utilize the DRS in their practice adhere to the guidelines provided in the **S1 Durham Risk Score Guide**.

Second, as detailed above, several DRS variables (e.g., abuse history) were not assessed during the NESARC Wave 1 assessment. We ultimately felt that the inclusion of these variables in the DRS was warranted, given that our explicit goal was to make the best possible clinical tool. Additionally, a sensitivity analysis revealed that the DRS continued to perform quite well when calculated exclusively from Wave 1 items; however, we fully recognize that this remains a limitation of the present work and that more work is still needed to verify the utility of these variables in studies where they are assessed prospectively.

Third, it is unclear how well the findings from the present research, which are derived entirely from participants who consented to participate in longitudinal research studies, might generalize to individuals undergoing clinical assessments or seeking treatment for mental health issues. One might expect that some individuals undergoing suicide risk evaluations would be less willing to disclose potential risk factors, including current and prior history of suicidal thoughts and behaviors. Of course, the latter concern also applies to virtually all clinical suicide risk assessments currently available, as nearly all such assessments rely on participants’ willingness to disclose suicidal thoughts and plans. Additionally, a strength of the DRS is that much of the score is derived from demographic and psychopathology-based risk factors, which should, theoretically, make it more robust to underreporting than traditional risk assessments that often rely exclusively on participants’ reports of suicidal thoughts and behaviors.

Fourth, we recognize that the DRS contains many items and may not be practical to adminster in some settings. For this reason, we are actively working to develop an abbreviated version of the measure. Fifth, although we are strong proponents of multiple imputation and maximum likelihood estimation methods to handle missingness in most situations, we elected to treat missing data as absent (i.e., “0”) in the calculation of DRS scores in this study because this approach best reflects real-world clinical practice. Further, we strongly believe that the benefits of a raw suicide attempt risk score that can be calculated and interpreted in virtually any clinical situation far outweigh the benefits of using more state-of-the-art approaches to handling missingness.

Finally, far more work is needed to develop more accurate short-term risk models (e.g., 1-week or 1-month models). Although the DRS is unable to accomplish this goal, we believe that identification of long-term suicide attempt risk is a key first step toward developing more accurate acute risk models. Specifically, consistent with fluid vulnerability theory [30] and diathesis–stress models more generally, we hypothesize that individuals who score highly on the DRS will have higher “set points” and will be more likely to attempt suicide when faced with highly stressful situations than individuals with lower scores. While such work was beyond the scope of the present study, we are hopeful that future prospective studies in this important area of inquiry will lead to improved prediction of acute risk, which, unfortunately, remains woefully inadequate at the present time.

### Clinical implications

Although clinicians must ultimately determine which treatments are most appropriate for their patients, consideration should be given to the idea of developing a safety plan [69] with any patient who scores ≥6 on the DRS (i.e., moderate-risk group status or higher), as this brief intervention has been shown to significantly reduce the occurrence of future suicidal behavior [70]. Moreover, as noted above, recent research by Ross and colleagues [66] indicates that the specificity (86%), sensitivity (82%) and PPV values (4%) corresponding to a cut score of 6 or higher on the DRS exceed the threshold accuracy values necessary for cost-effective implementation of suicide risk prediction with safety planning and follow-up. Consideration should also be given to ensuring that patients in the highest-risk groups have access to more intensive and long-term cognitive behavioral treatment approaches that have also been shown to reduce the occurrence of suicidal behavior [71–72]. Importantly, a cut score of 9 or greater on the DRS (corresponding to high-risk group status or approximately top 5% of scores) produces specificity (96%), sensitivity (58%), and PPV (10%) values that exceed the threshold accuracy values identified by Ross and colleagues [66] as necessary for suicide risk prediction to be combined with cognitive behavioral therapy for suicide prevention to become cost-effective from a healthcare sector perspective.

On the other hand, clinicians must also recognize that the DRS does not assess acute risk, as the latter determination requires in-depth review of current suicidal ideation, intent, plans, feasibility, access to means, and current stressors (among others). As such, the DRS should never be used as the sole basis to determine imminent suicide risk or need for civil commitment. Instead, individuals endorsing recent suicidal ideation or behaviors should always be assessed for intent and other indicators of acute risk not included in the DRS. Where possible, we recommend that the DRS be integrated with existing assessment and intake practices, as most DRS items are routinely assessed by mental health clinicians. Constructs not already assessed could likely be added with relatively little additional burden to mental health clinicians and clients. An additional advantage of making the DRS part of routine practice is that mandatory checklists have been shown to increase the occurrence of risk-appropriate treatment while simultaneously decreasing healthcare disparities [73]. Notably, there are documented disparities in the application of mental health and suicide risk assessments [74] that could potentially be eliminated with a DRS-based clinical decision support model.

A final clinical consideration concerns our finding that 42% of all prospective suicide attempts during the follow-up period occurred among individuals who reported no lifetime history of suicidal thoughts or behavior at baseline. This important finding speaks to the tremendous challenges that clinicians routinely face when attempting to stratify patients’ risk for future suicidal behavior, as the vast majority of current suicide risk screens primarily rely on patients’ current endorsement of suicidal thoughts and behaviors. In contrast, the approach taken in the development of the DRS has been to focus on a diverse array of longitudinal risk factors. Importantly, both the DRS total score and risk group status performed well among individuals with and without a lifetime history of suicidal thoughts or behaviors, which we believe provides strong support for our general approach to instrument development.

## Conclusions

In summary, our findings suggest that the DRS is a promising new, evidence-based approach to suicide attempt risk assessment. While more research is needed to prospectively evaluate this tool in independent samples and in clinical settings, our initial findings are encouraging and suggest that this novel approach has the potential to significantly enhance clinicians’ ability to identify individuals at risk for attempting suicide in the future.

### Disclaimers

The views expressed in this article are those of the authors and do not necessarily reflect the position or policy of the US Department of Veterans Affairs, the US Department of Defense, the US government, or any of the other affiliated institutions.

## Supporting information

S1 TRIPOD Checklist TRIPOD, Transparent Reporting of a multivariable prediction model for Individual Prognosis Or Diagnosis. (DOCX)

S1 Durham Risk Score Guide Suicide attempt risk checklist designed to assess patients’ risk for attempting suicide during the next 3 years. (DOCX)

S1 File Supporting information tables and figures. Table A: Bivariate AUC values and empirical evidence scores across the 3 development samples. Table B: Measures used to assess the constructs included in the DRS. Table C: Distribution, rates of suicide attempts, odds, and predicted probabilities by risk group status in total sample (*N* = 35,654). Table D: Association between AUC values and number of items assessed across samples. Table E: Summary of logistic regression conducted in the combined NESARC 1 and 2 development samples (*N* = 17,397). Table F: Items used to calculate the SAD PERSONS score in the NESARC study. Fig A: Association between total empirical evidence score and mean AUC value across the development samples. Fig B: Distribution of DRSs. Fig C: Distribution of DRSs among participants who attempted suicide during follow-up (*N* = 288). Fig D: Association between number of items and AUC values. AUC, area under the curve; DRS, Durham Risk Score; NESARC, National Epidemiologic Survey on Alcohol and Related Conditions.
(DOCX)

## Abbreviations

APA: American Psychiatric Association
AUC: area under the curve
AUDADIS: Alcohol Use Disorder and Associated Disabilities Interview Schedule
AUDIT: Alcohol Use Disorders Identification Test
BPD: borderline personality disorder
BSS: Beck Scale for Suicide Ideation
CAPS: Clinician-Administered PTSD Scale
CI: confidence interval
C-SSRS: Columbia-Suicide Severity Rating Scale
CTQ: Childhood Trauma Questionnaire
DAST: Drug Abuse Screening Test
DRS: Durham Risk Score
DTS: Davidson Trauma Scale
EHR: electronic health record
FN: false negative
FP: false positive
LEC: Life Events Checklist
LGBTQ: lesbian, gay, bisexual, transgender, and queer or questioning
MINI: Mini International Neuropsychiatric Interview
NESARC: National Epidemiologic Survey on Alcohol and Related Conditions
NPV: negative predictive value
NSSI: nonsuicidal self-injury
OR: odds ratio
PHQ-9: Patient Health Questionnaire-9
PPV: positive predictive value
PTSD: post-traumatic stress disorder
REHAB: Assessing and Reducing Post-Deployment Violence Risk
ROC: receiver operating characteristic
SBQ-R: Suicidal Behaviors Questionnaire-Revised
SCID: Structured Clinical Interview for DSM
SCL-90: Symptom Checklist-90
SHBQ: Self-Harm Behavior Questionnaire
SITBI: Self-Injurious Thoughts and Behaviors Interview
SPS: SAD PERSONS scale
TBI: traumatic brain injury
TLEQ: Traumatic Life Events Questionnaire
TN: true negative
TP: true positive
TRIPOD: Transparent Reporting of a multivariable prediction model for Individual Prognosis Or Diagnosis
VALOR: Veterans After-Discharge Longitudinal Registry
VR-12: Veterans Rand 12-Item Health Survey
