# Supplementary material for: Development and validation of the Durham Risk Score for estimating suicide attempt risk: A prospective cohort analysis
Source: PLoS Med. 2021 Aug 5;18(8):e1003713. doi: 10.1371/journal.pmed.1003713 (PMC8341885; doi:10.1371/journal.pmed.1003713)
Supplement: S1 Durham Risk Score Guide — (DOCX) [file pmed.1003713.s002.docx]

**S1 Durham Risk Score (DRS) Guide**

**Version 1.0**

Please use the Citation for the Primary Article when Citing the DRS

Kimbrel, N. A.,* Beckham, J. C., Calhoun, P. S., DeBeer, B. B., Keane, T. M., Lee, D. J., Marx, B. P., Meyer, E. C., Morissette, S. B., Rosen, R. C., & Elbogen, E. B. (2021). Development and validation of the Durham Risk Score for estimating suicide attempt risk: A prospective cohort analysis. *PLOS Medicine.*

* Correspondence concerning the Durham Risk Score should be sent to:

Dr. Nathan A. Kimbrel

Mid-Atlantic Mental Illness Research, Education, and Clinical Center (MIRECC)

Durham Veterans Affairs Health Care System

3022 Croasdaile Drive, Durham, NC, 27705

Email: [Nathan.Kimbrel@va.gov](mailto:Nathan.Kimbrel@va.gov)

Please note that the most up-to-date versions of the DRS and the DRS Guide will be provided at the following website [**https://www.mirecc.va.gov/visn6/Providers.asp**](https://www.mirecc.va.gov/visn6/Providers.asp) following publication of this article along with a web-based calculator to facilitate rapid scoring of the instrument.

**Overview**

The Durham Risk Score (DRS), Version 1.0 is a suicide attempt risk checklist designed to assess patients’ risk for attempting suicide during the next 3 years. It is freely available to clinicians, administrators, and researchers for non-commercial purposes. Clinicians can calculate the DRS total score, suicide attempt risk group assignment, and predicted probability of attempting suicide by hand using **S1 Table C**. The most up-to-date versions of the DRS Checklist and DRS Guide will be placed at the Mid-Atlantic MIRECC Clinical Core’s webpage at: [**https://www.mirecc.va.gov/visn6/Providers.asp**](https://www.mirecc.va.gov/visn6/Providers.asp). Clinicians, researchers, and others who use the DRS are required to cite the original source article (i.e., Kimbrel et al., 2021) when referencing the DRS in publications and other communications. We also plan to create an online calculator to accompany the measure which will also be provided at the Mid-Atlantic MIRECC Clinical Core’s webpage at: [**https://www.mirecc.va.gov/visn6/Providers.asp**](https://www.mirecc.va.gov/visn6/Providers.asp).

***Basic Scoring Instructions for the Durham Risk Score (DRS) Checklist, Version 1.0***

To calculate the DRS Version 1.0 total score using the checklist format, simply score and sum the 23 items from the DRS to calculate the total score. Total scores can range from 0-27. Items that are not present or are have not been assessed are always coded as “0”. The majority of items (n=19) are scored as “1” when present; however, **please note that 4 of the 23 DRS items (item 1: lifetime suicide attempts; item 4: lifetime NSSI; item 6: lifetime psychiatric hospitalization; and item 7; lifetime borderline personality disorder) are scored as “2” when present because they are particularly strong risk factors for suicide attempts** [3]. To determine which risk group a patient should be assigned to, consult **S1 Table C**. This table can also be used to identify patients’ predicted probability of attempting suicide in the next 3 years based on risk group assignment. These probabilities were derived from a logistic regression model that included all 35,654 participants and analyzed risk group status as a categorical predictor of prospective attempts [*Χ*^2^ (5) *=* 921.0, *p* < .001, Nagelkerke *R*^2^ = .28]. **S1 Table C** also reports odds ratios for each risk group (where the lowest risk group is the referent category) across the total sample (*N*=35,654).

***The Durham Risk Score Should NOT Be Used to Make Determinations of Acute Risk for Suicide***

Please note that the DRS should never be used as the sole basis for a determination of a patient’s acute risk for suicide. *The DRS was not designed to assess acute/imminent suicide risk, and there is currently no evidence that it will be useful in such situations.* Instead, clinicians making determinations regarding acute suicide risk should gather as much information as possible regarding patients’ current suicidal ideation, intent, plans, feasibility, access to means, and current stressors (among others) and use their clinical judgement—in consultation with that of other trained professionals—to determine the patient’s current level of acute risk. Thus, the DRS should not be used as the sole basis for a civil commitment, nor should it be used as the sole basis for a suicide risk assessment, as patients that endorse recent ideation or behaviors should always be assessed for current intent and other indicators of acute risk not included in the DRS. Thus, we view the DRS as complementary to clinicians’ existing practices and recommend that the DRS be integrated with existing assessments and evaluation practices, as the majority of constructs included in the score are routinely assessed by mental health clinicians. Constructs not already assessed during intake evaluations could likely be added with relatively little additional burden to clinicians and clients.

***Minimum Number of Items Necessary to Use the Durham Risk Score***

*We recommend that clinicians systematically assess and score all 23 items using the most reliable and valid assessment methods available to them at the time of the assessment*. In the event that an item cannot be readily assessed by the clinician, or if the clinician is unsure of how to code an item, then that item should be scored as “0” and not counted toward the total score. *If clinicians are unable to systematically assess and score at least 21 of the 23 items [including all four of the double-weighted items (i.e., item 1: lifetime suicide attempts; item 4: lifetime NSSI; item 6: lifetime psychiatric hospitalization; and item 7; lifetime borderline personality disorder), then we recommend against using the DRS*, as we have observed a highly-significant positive association (*r* = .94, *p* = .006) between number of items assessed and AUC values (**S1 Fig D**). We have further observed that the DRS performed best (overall AUC = .91) in the four samples that systematically assessed at least 21 of the 23 items, including item 1: lifetime suicide attempts; item 4: lifetime NSSI; item 6: lifetime psychiatric hospitalization; and item 7; lifetime borderline personality disorder).

***Importance of Using Reliable, Valid, and Standardized Assessment Methods***

While our initial findings concerning the predictive validity of the DRS are encouraging, additional prospective research is still needed to validate the DRS in independent samples. Research is also still needed to determine how best to assess each of the 23 constructs used to calculate the DRS score. Moreover, while we believe that the present findings provide strong empirical support for the DRS and the 23 constructs used to calculate the score, more work is needed to determine how best to assess each of these constructs. Additionally, *it is particularly important to note that there is presently no empirical support for the DRS in situations in which clinicians rely exclusively on their clinical impressions of patients to calculate the DRS score* (as opposed to using standardized instruments to assess each of the 23 DRS constructs). Thus, while we think that it is reasonable for clinicians who use similar high-quality instruments to assess complex DRS constructs (e.g., psychiatric disorders) to expect the instrument to perform well in their own clinics, we would not expect the instrument to perform as well in situations in which clinicians rely exclusively upon their clinical judgement and/or unstructured interviews to assess all DRS items (particularly the more complex constructs). It may be the case that future studies determine that such approaches are acceptable; however, at the present time, we only have data derived from secondary analyses of high-quality research studies that typically used reliable and valid instruments to assess the constructs of interest to support the validity of the DRS. As such, *we recommend that clinicians always strive to use the most reliable, valid, and standardized assessment methods available to them at the time of the evaluation.*

***General Scoring Procedures for Items Assessed in Both Past Year and Lifetime Timeframes***

For all items that are assessed for both past 12 months and lifetime, any item scored as present during the past 12 months should be automatically coded as present for lifetime as well. Note that the online DRS calculator will automatically score any item present during the past 12 months as also being present during lifetime.

***Scoring Notes for Specific Items on the DRS Version 1.0***

***Item 1.*** Patients who have made an actual suicide attempt at any point in their lifetime should be scored as **“2”**, whereas patients who have never attempted suicide should be should be scored as “0”. **Note that this item is one of four items scored as present = 2.** Patients who have made aborted [36] or interrupted [36] suicide attempts, but who have never made an actual suicide attempt during their lifetime should also be scored as “0”. If this item was not assessed, or if clinicians are unsure of how to code the item, it should be scored as “0”. Additionally, if a patient reports a lifetime history of suicide attempts, then item 3 (suicidal ideation, lifetime) should also be automatically coded as present = 1, regardless of the patients’ response. Thus, if a patient reports a lifetime suicide attempt, but denies having had any type of thought about suicide prior to making the attempt, the clinician should still score lifetime suicidal ideation (item 3) as present to ensure that any patient who has attempted suicide during their lifetime has a minimum score of 3 (i.e., 2 + 1 = 3). We recommend that a validated structured clinical interview (e.g., SITBI, C-SSRS) be used to assess suicide attempts whenever possible. Note that the online DRS Calculator will automatically score item 1 as present=2. It will also automatically score item 3 (lifetime suicidal ideation) as present if item 1 (lifetime suicide attempts) is scored as present.

***Item 2.*** Patients who have had thoughts of killing themselves or have experienced any other type of ideation (including wishing to be dead or wishing to fall asleep and to not wake up) in the past 12 months should be scored as “1”, whereas patients who have not not experienced any type of suicidal ideation in the past 12 months should be should be scored as “0”. If this item was not assessed, or if clinicians are unsure of how to code the item, it should be scored as “0”. Additionally, if a patient reports suicidal ideation in the past 12 months on item 2, then item 3 (lifetime suicidal ideation) should be automatically coded as present = 1. We recommend that validated structured clinical interviews (e.g., SITBI, C-SSRS) be used to assess suicidal ideation whenever possible. Note that the online DRS Calculator will automatically score item 3 (lifetime ideation) as present if item 2 (current ideation) is scored as present by the clinician.

***Item 3.*** Patients who have had thoughts of killing themselves or have experienced any other type of suicidal ideation (including wishing to be dead or wishing to fall asleep and to not wake up) at any point during their lifetime should be scored as “1”, whereas patients who have not not experienced any type of suicidal ideation during their lifetime should be should be scored as “0”. If this item was not assessed, or if clinicians are unsure of how to code the item, it should be scored as “0. We recommend that validated structured clinical interviews (e.g., SITBI, C-SSRS) be used to assess suicidal ideation whenever possible. Patients who have previously made a suicide attempt (item 1) or who are currently experiencing suicidal ideation (item 2) should always be coded as having lifetime ideation (item 3). Note that the DRS Calculator will automatically score item 3 as present if item 1 or 2 is marked as present.

***Item 4.*** Patients who have have engaged in any type of nonsuicidal self-injury (i.e., NSSI), such as intentionally cutting, burning, scratching, or hitting themselves for nonsuicidal reasons, during their lifetime should be scored as “**2**”, whereas patients who have not should be should be scored as “0”. If this item was not assessed, or if clinicians are unsure of how to code the item, it should be scored as “0”. **Note that this item is one of four items scored as present = 2.** Thus, a patient who reports a lifetime history of NSSI will have a minimum score of 2. We recommend that a validated NSSI instrument that asssesses a wide range of NSSI behaviors (e.g., SITBI) be used to assess NSSI. Note that the DRS calculator will automatically score item 4 as present=2.

***Item 5.*** Patients who have been hospitalized overnight for a psychiatric or substance use problem during the past 12 months should be scored as “1”, whereas patients who have not been hospitalized overnight for a psychiatric or substance use problem in the past 12 months should be scored as “0”. If this item was not assessed, or if clinicians are unsure of how to code the item, it should be scored as “0”. Additionally, if a patient reports having been hospitalized overnight for a psychiatric or substance use problem in the past 12 months, then item 6 (lifetime hospitalization) should be automatically coded as present = 2. Thus, a patient who reports a history of psychiatric hospitalization in the past 12 months will have a minimum score of 3. Note that the online DRS calculator will automatically score item 6 as present=2 if item 5 is scored as present.

***Item 6.*** Patients who have have been hospitalized overnight for a psychiatric or substance use problem at any point during their lifetime should be scored as “**2**”, whereas patients who have never been hospitalized overnight for a psychiatric or substance use problem during their lifetime should be should be scored as “0”. If this item was not assessed, or if clinicians are unsure of how to code the item, it should be scored as “0”. **Note that this item is one of four items scored as present = 2.** Thus, a patient who reports a lifetime history of psychiatric hospitalization will have a minimum score of 2. Note that the DRS calculator will automatically score item 6 as present=2.

***Item 7.*** Patients who meet lifetime criteria for borderline personality disorder should be scored as “**2**”, whereas patients who do not meet lifetime criteria for borderline personality disorder should be scored as “0”. If this item was not assessed, or if clinicians are unsure of how to code the item, it should be scored as “0”. **Note that this item is one of four items scored as present = 2.** Thus, a patient who meets full diagnostic criteria for lifetime borderline personality disorder will have a minimum score of 2. We recommend that a validated structured clinical interview (e.g., SCID, DIP-D) be used to assess borderline personality disorder whenever possible.

***Item 8.*** Patients who meet lifetime diagnostic criteria for bipolar disorder, depression, or dysthymia should be coded as “1”, whereas patients who do not meet lifetime criteria for any of these disorders should be coded as “0”. If these disorders were not assessed, or if clinicians are unsure of how to code the item, it should be scored as “0”. We recommend that validated clinical interviews (e.g., SCID) be used to assess these disorders whenever possible.

***Item 9.*** Patients who currently meet full diagnostic criteria for posttraumatic stress disorder (PTSD) should receive a score of “1”, whereas patients who do not currently meet full diagnostic criteria for PTSD should receive a score of “0”. If PTSD was not assessed, or if clinicians are unsure of how to code the item, it should be scored as “0”. Note that patients who previously met full diagnostic criteria for PTSD, but no longer do at the time of the assessment should be scored as absent = 0 (i.e., PTSD in partial remission should not be counted as meeting criteria for this item). We recommend that a validated clinical interview (e.g., CAPS, SCID) be used to assess PTSD whenever possible.

***Item 10.*** Patients who currently meet diagnostic criteria for a substance use disorder (SUD) in the past 12 months, including alcohol use disorder, cannabis use disorder, hallucinogen use disorder, inhalant use disorder, opioid use disorder, sedative/hypontic/anxiolytic use disorder, or stimulant use disorder, should receive a score of “1”. Note that caffeine- and tobacco-related disorders should *not* be counted toward meeting this criterion. Patients that meet the specifier of “in early remission” should also be scored as present = 1, because they have continued to report clinically-significant SUD symptoms within the past 12 months. In contrast, patients who meet criteria for the “In sustained remission” specifier (i.e., no SUD symptoms have been present during the past 12 months except craving) should be scored as “0” since no symptoms were present during the past 12 months. In DSM-IV parlance, only SUD patients who meet the “In full substained remission” specifier should be scored as “0”; all other DSM-IV specifiers (i.e., “In substained partial remission”, “In early full remission”, and “In early partial remission”) should be coded as “1”. If SUDs were not assessed, or if clinicians are unsure of how to code the item, it should be scored as “0”. We recommend that a validated clinical interview (e.g., SCID) be used to assess SUD whenever possible.

***Item 11.*** Patients who report engaging in binge drinking one or more times per week on average over the past 12 months should be coded as “1”, whereas patients who either do not report binge drinking or else report binge drinking at a frequency less than weekly for the past 12 months should be coded as “0”. If frequency and quantity of drinking was not assessed, or if clinicians are unsure of how to code the item, it should be scored as “0”. Information regarding patients’ quantity and frequency of drinks per week can be obtained as part of a validated interview for alcohol use disorder (e.g., SCID-5) or from a validated self-report instument (e.g., AUDIT, although note that item 3 from the AUDIT actually asks how often patients have had 6 or more drinks on a single occasion, which is a higher quantity for both men and women than the current NIAAA guidelines for binge drinking).

***Item 12.*** Patients who report a lifetime history of being incarcerated or who have a history of physical violence towards others or should be coded as “1”. Patients with no lifetime history of violent behavior or incarceration should be coded as “0”. If history of violence and incarceration were not assessed, or if clinicians are unsure of how to code the item, it should be scored as “0”.

***Item 13.*** Patients who report currently smoking cigarettes or cigars should be coded as “1”. Never and former smokers should be coded as “0”. If smoking status was not assessed, or if clinicians are unsure of how to code the item, it should be scored as “0”. We recommend that clinicians use a single, validated item to assess smoking status (e.g., from the FTND). In addition, note that none of the studies included in the cohorts used to develop the DRS assessed for vaping, nor are we aware of any definitive evidence to date that vaping is associated with increased risk for suicide attempts. Accordingly, at the present time, we recommend that vaping alone (i.e., in the absence of any other smoking behavior) be coded as “0”.

***Item 14.*** Patients who report that they have been “extremely” bothered by difficulties related to either staying or falling asleep in the past month should be coded as “1”. Patients who report less severe sleep problems (i.e., any response less than the most extreme response possible on a given scale) should be coded as absent=0. If sleep problems were not assessed, or if clinicians are unsure of how to code the item, it should be scored as “0”. We recommend that a single, Likert-based item from a validated self-report instrument of either PTSD or depression (e.g., PCL-5 or PHQ-9, respectively) be used to assess extreme sleep difficulties.

***Item 15.*** Patients who report personally experiencing a lifetime history of being sexually abused as a child or sexually assaulted as an adult should be coded as “1”, whereas patients who report no lifetime history of sexual abuse or assault should be coded as “0”. If history of sexual abuse and assault were not assessed, or if clinicians are unsure of how to code the item, it should be scored as “0”. We recommend that a validated measure of traumatic exposure (e.g., TLEQ , CTQ , LEC) be used to assess history of sexual abuse and assault whenever possible.

***Item 16.*** Patients who report personally experiencing physical abuse as a child (i.e., prior to the age of 18) should be coded as “1”, whereas patients who report no lifetime history of personally experiencing childhood physical abuse should be coded as “0”. Patients who only report a history of physical assault as adults, or who report having witnessed family violence but were not actually physically assaulted themselves, should also be coded as absent=0. If history of childhood physical abuse was not assessed, or if clinicians are unsure of how to code the item, it should be scored as “0”. We recommend that a validated measure of traumatic exposure (e.g., TLEQ , CTQ, LEC) be used to assess this construct whenever possible.

***Item 17.*** Lower income patients (i.e., patients with an annual household income less than 2/3 the national median annual household income [68]) should be coded as “1”. Middle- to upper income patients (i.e., patients whose annual household income is greater than, or equal to, 2/3 national median household income) should be coded as “0”. If household income was not assessed, or if clinicians are unsure of how to code this item, it should be scored as “0”. We recommend that clinicians in the U.S. use the definition of less than two-thirds the U.S. median household income to define lower income based on current U.S. Census Data. At the time of the present analyses, this resulted in lower income being operationalized as < $40,000 per year ($59,039 x .67 = $39,557). For clinicians outside of the U.S., we recommend that they use their best judgement to determine if participants are of lower income relative to the majority of individuals in their community and/or are experiencing significant financial hardships.

***Item 18.*** Patients who are currently unemployed should be scored as “1”, whereas patients who are employed full time or part-time, disabled, retired, are a full-time student, or are a full-time homemaker should be scored as “0”. If employment status was not assessed, or if clinicians are unsure of how to code this item, it should be scored as “0”.

***Item 19.*** Patients who identify as gay, lesbian, bisexual, transgendered, or questioning (LGBTQ) should be coded as “1”, whereas patients who identify as heterosexual and cisgendered should be coded as “0”. If sexual orientation and gender identity were not assessed, or if clinicians are unsure of how to code this item, it should be scored as “0”.

***Item 20.*** Patients assigned female sex at birth should be coded as “1”, whereas patients assigned male sex at birth should be coded as “0”. If this item was not assessed, or if clinicians are unsure of how to code the item, it should be scored as “0”.

***Item 21.*** Patients under the age of 35 years of age should be coded as “1”, whereas patients who are 35 years or older should be coded as “0”. If this item was not assessed, or if clinicians are unsure of how to code the item, it should be scored as “0”.

***Item 22.*** Patients who did not receive a high school diploma, GED, or high school equivalency and are not still in high school working toward graduation should be scored as “1”. Individuals who have received a high school diploma, GED, high school equivalency, or who are still in high school working toward their diploma (e.g., an 18-year old high school student) should be scored as “0”. If this item was not assessed, or if clinicians are unsure of how to code the item, it should be scored as “0”.

***Item 23.*** Patients who rate their general health to be only “fair” or “poor” should be scored as “1”, whereas patients who rate their general health to be “excellent”, “very good”, or “good” should be scored as “0”. If this item was not assessed, or if clinicians are unsure of how to code the item, it should be scored as “0”. We recommend that clinicians use a single, Likert-based item from a validated measure (e.g,., VR-12) to assess this construct.
