# Supplementary material for: Development and validation of the Durham Risk Score for estimating suicide attempt risk: A prospective cohort analysis
Source: PLoS Med. 2021 Aug 5;18(8):e1003713. doi: 10.1371/journal.pmed.1003713 (PMC8341885; doi:10.1371/journal.pmed.1003713)
Supplement: S1 File — Table A: Bivariate AUC values and empirical evidence scores across the 3 development samples. Table B: Measures used to assess the constructs included in the DRS. Table C: Distribution, rates of suicide attempts, odds, and predicted probabilities by risk group status in total sample (N = 35,654). Table D: Association between AUC values and number of items assessed across samples. Table E: Summary of logistic regression conducted in the combined NESARC 1 and 2 development samples (N = 17,397). Table F: Items used to calculate the SAD PERSONS score in the NESARC study. Fig A: Association between total empirical evidence score and mean AUC value across the development samples. Fig B: Distribution of DRSs. Fig C: Distribution of DRSs among participants who attempted suicide during follow-up (N = 288). Fig D: Association between number of items and AUC values. AUC, area under the curve; DRS, Durham Risk Score; NESARC, National Epidemiologic Survey on Alcohol and Related Conditions. (DOCX) [file pmed.1003713.s003.docx]

**Table A**

***Bivariate Area Under the Curve (AUC) Values and Empirical Evidence Scores Across the Three Development Samples.***

| **Variable** | **Available for Analysis** | **Durham Risk Score** | **SAD PERSONS** | **Broad Risk Category** | **Evidence for Category for Suicide Deaths [3]** | **Evidence for Category for Attempts [3]** | **Evidence for Variable for Suicide Deaths [3]** | **Evidence for Variable for Attempts [3]** | **Total Empirical Evidence Score** | **% of Development Samples Containing Variable** | **NESARC 1 AUC** | **NESARC 2 AUC** | **REHAB AUC** | **Mean AUC Across Development Samples** |
| --- | --- | --- | --- | --- | --- | --- | --- | --- | --- | --- | --- | --- | --- | --- |
| Attempt (Past Month) |  |  |  | Prior SITBIs | 7 | 10 | 9 | 9 | 35 | 0% | X | X | X | X |
| Attempt (Past Year) |  |  |  | Prior SITBIs | 7 | 10 | 9 | 9 | 35 | 0% | X | X | X | X |
| **Attempt (Lifetime)** | **** | **** | **** | **Prior SITBIs** | **7** | **10** | **9** | **9** | **35** | **100%** | **0.70** | **0.62** | **0.53** | **0.62** |
| Hospitalization (Past Month) |  |  |  | Treatment History | 10 | 5 | 10 | 6 | 31 | 0% | X | X | X | X |
| **Hospitalization (Past Year)** | **** | **** | **** | **Treatment History** | **10** | **5** | **10** | **6** | **31** | **67%** | **0.52** | **0.53** | X | **0.52** |
| **Hospitalization (Lifetime)** | **** | **** | **** | **Treatment History** | **10** | **5** | **10** | **6** | **31** | **100%** | **0.68** | **0.65** | **0.64** | **0.66** |
| NSSI (Past Month) |  |  |  | Prior SITBIs | 7 | 10 | 0 | 10 | 27 | 0% | X | X | X | X |
| NSSI (Past Year) |  |  |  | Prior SITBIs | 7 | 10 | 0 | 10 | 27 | 0% | X | X | X | X |
| **NSSI (Lifetime)** | **** | **** | ** ** | **Prior SITBIs** | **7** | **10** | **0** | **10** | **27** | **100%** | **0.67** | **0.69** | **0.72** | **0.69** |
| Ideation (Past Month) |  |  |  | Prior SITBIs | 7 | 10 | 8 | 0 | 25 | 33% | X | X | 0.67 | 0.67 |
| **Ideation (Past Year)** | **** | **** | **** | **Prior SITBIs** | **7** | **10** | **8** | **0** | **25** | **33%** | X | X | **0.67** | **0.67** |
| **Ideation (Lifetime)** | **** | **** | **** | **Prior SITBIs** | **7** | **10** | **8** | **0** | **25** | **100%** | **0.76** | **0.65** | **0.75** | **0.72** |
| **Borderline PD (Lifetime)** | **** | **** | **** | **Psychopathology** | **8** | **8** | **0** | **7** | **23** | **67%** | **0.79** | **0.74** | X | **0.77** |
| Avoidant PD (Lifetime) |  |  |  | Psychopathology | 8 | 8 | 0 | 7 | 23 | 67% | 0.55 | 0.54 | X | 0.55 |
| Dependent PD (Lifetime) |  |  |  | Psychopathology | 8 | 8 | 0 | 7 | 23 | 67% | 0.53 | 0.52 | X | 0.53 |
| OCPD (Lifetime) |  |  |  | Psychopathology | 8 | 8 | 0 | 7 | 23 | 67% | 0.56 | 0.52 | X | 0.54 |
| Paranoid PD (Lifetime) |  |  |  | Psychopathology | 8 | 8 | 0 | 7 | 23 | 67% | 0.60 | 0.58 | X | 0.59 |
| Schizoid PD (Lifetime) |  |  |  | Psychopathology | 8 | 8 | 0 | 7 | 23 | 67% | 0.58 | 0.53 | X | 0.55 |
| Histrionic PD (Lifetime) |  |  |  | Psychopathology | 8 | 8 | 0 | 7 | 23 | 67% | 0.54 | 0.54 | X | 0.54 |
| Plan (Past Month) |  |  |  | Prior SITBIs | 7 | 10 | 0 | 0 | 17 | 0% | X | X | X | X |
| Plan (Past Year) |  |  |  | Prior SITBIs | 7 | 10 | 0 | 0 | 17 | 0% | X | X | X | X |
| Plan (Lifetime) |  |  |  | Prior SITBIs | 7 | 10 | 0 | 0 | 17 | 0% | X | X | X | X |
| Intent (Past Month) |  |  |  | Prior SITBIs | 7 | 10 | 0 | 0 | 17 | 0% | X | X | X | X |
| Intent (Past Year) |  |  |  | Prior SITBIs | 7 | 10 | 0 | 0 | 17 | 0% | X | X | X | X |
| Intent (Lifetime) |  |  |  | Prior SITBIs | 7 | 10 | 0 | 0 | 17 | 0% | X | X | X | X |
| Suicide Screening Instrument |  |  |  | Screening Instrument | 0 | 8 | 0 | 8 | 16 | 0% | X | X | X | X |
| SLE: Financial Problems |  |  |  | Social Factors | 6 | 1 | 6 | 0 | 13 | 67% | 0.64 | 0.62 | X | 0.63 |
| SLE: Divorce/Break Up |  |  |  | Social Factors | 6 | 1 | 6 | 0 | 13 | 67% | 0.60 | 0.61 | X | 0.60 |
| **SLE: Unemployed** | **** | **** | **** | **Social Factors** | **6** | **1** | **6** | **0** | **13** | **100%** | **0.62** | **0.57** | **0.54** | **0.58** |
| SLE: Change in Work/Hrs. |  |  |  | Social Factors | 6 | 1 | 6 | 0 | 13 | 67% | 0.62 | 0.55 | X | 0.58 |
| SLE: Fired or Laid Off |  |  |  | Social Factors | 6 | 1 | 6 | 0 | 13 | 67% | 0.57 | 0.55 | X | 0.56 |
| SLE: Social Problems |  |  |  | Social Factors | 6 | 1 | 6 | 0 | 13 | 67% | 0.58 | 0.59 | X | 0.58 |
| SLE: Boss/Coworker Problems (Past Year) |  |  |  | Social Factors | 6 | 1 | 6 | 0 | 13 | 67% | 0.58 | 0.54 | X | 0.56 |
| SLE: Legal Problems |  |  |  | Social Factors | 6 | 1 | 6 | 0 | 13 | 67% | 0.56 | 0.52 | X | 0.54 |
| SLE: Victim of Crime |  |  |  | Social Factors | 6 | 1 | 6 | 0 | 13 | 67% | 0.56 | 0.52 | X | 0.54 |
| **Poor Perceived Health** | **** | **** | **** | **Physical Illness** | **5** | **6** | **0** | **0** | **11** | **67%** | **0.63** | **0.61** | X | **0.62** |
| Physical Illness (Any) | **** | **** | **** | Physical Illness | 5 | 6 | 0 | 0 | 11 | **67%** | 0.57 | 0.53 | X | 0.55 |
| Arteriosclerosis | **** | **** | **** | Physical Illness | 5 | 6 | 0 | 0 | 11 | **67%** | 0.50 | 0.51 | X | 0.51 |
| Blood Pressure/Hypertension | **** | **** | **** | Physical Illness | 5 | 6 | 0 | 0 | 11 | **67%** | 0.48 | 0.50 | X | 0.49 |
| Cirrhosis | **** | **** | **** | Physical Illness | 5 | 6 | 0 | 0 | 11 | **67%** | 0.50 | 0.51 | X | 0.50 |
| Other Liver Disease | **** | **** | **** | Physical Illness | 5 | 6 | 0 | 0 | 11 | **67%** | 0.53 | 0.51 | X | 0.52 |
| Chest Pain/Angina | **** | **** | **** | Physical Illness | 5 | 6 | 0 | 0 | 11 | **67%** | 0.53 | 0.51 | X | 0.52 |
| Tachycardia | **** | **** | **** | Physical Illness | 5 | 6 | 0 | 0 | 11 | **67%** | 0.56 | 0.51 | X | 0.53 |
| Heart Attack | **** | **** | **** | Physical Illness | 5 | 6 | 0 | 0 | 11 | **67%** | 0.51 | 0.50 | X | 0.50 |
| Heart Disease | **** | **** | **** | Physical Illness | 5 | 6 | 0 | 0 | 11 | **67%** | 0.51 | 0.50 | X | 0.51 |
| Stomach Ulcer | **** | **** | **** | Physical Illness | 5 | 6 | 0 | 0 | 11 | **67%** | 0.56 | 0.53 | X | 0.55 |
| Gastritis | **** | **** | **** | Physical Illness | 5 | 6 | 0 | 0 | 11 | **67%** | 0.55 | 0.52 | X | 0.53 |
| Arthritis | **** | **** | **** | Physical Illness | 5 | 6 | 0 | 0 | 11 | **67%** | 0.52 | 0.53 | X | 0.53 |
| Income < $20,000 |  |  |  | Demographics | 3 | 0 | 7 | 0 | 10 | 67% | 0.58 | 0.59 | X | 0.59 |
| **Income < $40,000** | **** | **** | **** | **Demographics** | **3** | **0** | **7** | **0** | **10** | **67%** | **0.58** | **0.61** | X | **0.59** |
| Income < $60,000 |  |  |  | Demographics | 3 | 0 | 7 | 0 | 10 | 67% | 0.55 | 0.54 | X | 0.55 |
| Income < $80,000 |  |  |  | Demographics | 3 | 0 | 7 | 0 | 10 | 67% | 0.54 | 0.52 | X | 0.53 |
| **Physical Abuse** | **** | **** | **** | **Social Factors** | **6** | **1** | **0** | **0** | **7** | **100%** | **0.61** | **0.60** | **0.60** | **0.60** |
| **Sexual Abuse/Assault** | **** | **** | **** | **Social Factors** | **6** | **1** | **0** | **0** | **7** | **100%** | **0.70** | **0.68** | **0.55** | **0.64** |
| Social Supports Lacking | **** | **** | **** | Social Factors | 6 | 1 | 0 | 0 | 7 | 67% | 0.65 | 0.59 | X | 0.62 |
| **Severe Sleep Problems** | **** | **** | **** | **Internalizing** | **2** | **4** | **0** | **0** | **6** | **33%** | X | X | **0.72** | **0.72** |
| **PTSD (Past Year)** | **** | **** | **** | **Internalizing** | **2** | **4** | **0** | **0** | **6** | **100%** | **0.66** | **0.64** | **0.65** | **0.65** |
| PTSD (Lifetime) | **** | **** | **** | Internalizing | 2 | 4 | 0 | 0 | 6 | 100% | 0.70 | 0.63 | 0.62 | 0.65 |
| **Mood Disorder (Lifetime)** | **** | **** | **** | **Internalizing** | **2** | **4** | **0** | **0** | **6** | **100%** | **0.74** | **0.64** | **0.74** | **0.70** |
| Mood Disorder (Past Year) |  |  |  | Internalizing | 2 | 4 | 0 | 0 | 6 | 100% | 0.68 | 0.67 | 0.83 | 0.73 |
| Major Depression (Past Year) | **** | **** | **** | Internalizing | 2 | 4 | 0 | 0 | 6 | 100% | 0.66 | 0.64 | 0.76 | 0.69 |
| Major Depression (Lifetime) | **** | **** | **** | Internalizing | 2 | 4 | 0 | 0 | 6 | 100% | 0.73 | 0.62 | 0.69 | 0.68 |
| Dysthymia (Past Year) | **** | **** | **** | Internalizing | 2 | 4 | 0 | 0 | 6 | 100% | 0.60 | 0.62 | 0.48 | 0.57 |
| Dysthymia (Lifetime) | **** | **** | **** | Internalizing | 2 | 4 | 0 | 0 | 6 | 100% | 0.65 | 0.64 | 0.48 | 0.59 |
| Manic/Bipolar I (Past Year) | **** | **** | **** | Internalizing | 2 | 4 | 0 | 0 | 6 | 100% | 0.54 | 0.58 | 0.59 | 0.57 |
| Manic/Bipolar I (Lifetime) | **** | **** | **** | Internalizing | 2 | 4 | 0 | 0 | 6 | 100% | 0.56 | 0.61 | 0.53 | 0.57 |
| Hypomanic/Bipolar II (Past Year) | **** | **** | **** | Internalizing | 2 | 4 | 0 | 0 | 6 | 100% | 0.51 | 0.49 | 0.50 | 0.50 |
| Hypomanic/Bipolar II (Lifetime) | **** | **** | **** | Internalizing | 2 | 4 | 0 | 0 | 6 | 100% | 0.51 | 0.50 | 0.50 | 0.50 |
| Anxiety Disorder (Past Year) | **** | **** | **** | Internalizing | 2 | 4 | 0 | 0 | 6 | 100% | 0.61 | 0.58 | 0.57 | 0.59 |
| Anxiety Disorder (Lifetime) | **** | **** | **** | Internalizing | 2 | 4 | 0 | 0 | 6 | 100% | 0.63 | 0.58 | 0.62 | 0.61 |
| Panic Disorder (Past Year) | **** | **** | **** | Internalizing | 2 | 4 | 0 | 0 | 6 | 100% | 0.58 | 0.56 | 0.55 | 0.56 |
| Panic Disorder (Lifetime) | **** | **** | **** | Internalizing | 2 | 4 | 0 | 0 | 6 | 100% | 0.61 | 0.57 | 0.59 | 0.59 |
| Agoraphobia (Past Year) | **** | **** | **** | Internalizing | 2 | 4 | 0 | 0 | 6 | 100% | 0.54 | 0.53 | 0.50 | 0.52 |
| Agoraphobia (Lifetime) | **** | **** | **** | Internalizing | 2 | 4 | 0 | 0 | 6 | 100% | 0.54 | 0.54 | 0.50 | 0.53 |
| Social Phobia (Past Year) | **** | **** | **** | Internalizing | 2 | 4 | 0 | 0 | 6 | 100% | 0.53 | 0.54 | 0.49 | 0.52 |
| Social Phobia (Lifetime) | **** | **** | **** | Internalizing | 2 | 4 | 0 | 0 | 6 | 100% | 0.53 | 0.54 | 0.49 | 0.52 |
| Specific Phobia (Past Year) | **** | **** | **** | Internalizing | 2 | 4 | 0 | 0 | 6 | 100% | 0.54 | 0.52 | 0.53 | 0.53 |
| Specific Phobia (Lifetime) | **** | **** | **** | Internalizing | 2 | 4 | 0 | 0 | 6 | 100% | 0.56 | 0.51 | 0.53 | 0.53 |
| GAD (Past Year) | **** | **** | **** | Internalizing | 2 | 4 | 0 | 0 | 6 | 100% | 0.57 | 0.57 | 0.50 | 0.55 |
| GAD (Lifetime) | **** | **** | **** | Internalizing | 2 | 4 | 0 | 0 | 6 | 100% | 0.59 | 0.57 | 0.50 | 0.55 |
| Loss of Rational Thinking | **** | **** | **** | Internalizing /Psychosis | 2 | 4 | 0 | 0 | 6 | 100% | 0.59 | 0.63 | 0.52 | 0.58 |
| **Violence/Incarceration** | **** | **** | **** | **Externalizing** | **4** | **0** | **0** | **0** | **4** | **100%** | **0.65** | **0.61** | **0.46** | **0.57** |
| **Weekly Binge Drinking** | **** | **** | **** | **Externalizing** | **4** | **0** | **0** | **0** | **4** | **100%** | **0.53** | **0.51** | **0.56** | **0.53** |
| **Current Smoker** | **** | **** | **** | **Externalizing** | **4** | **0** | **0** | **0** | **4** | **100%** | **0.66** | **0.59** | **0.52** | **0.59** |
| **Any Substance (Past Year)** | **** | **** | **** | **Externalizing** | **4** | **0** | **0** | **0** | **4** | **100%** | **0.59** | **0.55** | **0.63** | **0.59** |
| Any Substance (Lifetime) |  |  |  | Externalizing | 4 | 0 | 0 | 0 | 4 | 100% | 0.61 | 0.57 | 0.44 | 0.54 |
| Alcohol (Past Year) |  |  |  | Externalizing | 4 | 0 | 0 | 0 | 4 | 100% | 0.57 | 0.55 | 0.63 | 0.58 |
| Alcohol (Lifetime) |  |  |  | Externalizing | 4 | 0 | 0 | 0 | 4 | 100% | 0.57 | 0.52 | 0.47 | 0.52 |
| Stimulant (Past Year) |  |  |  | Externalizing | 4 | 0 | 0 | 0 | 4 | 100% | 0.51 | 0.50 | 0.50 | 0.50 |
| Stimulant (Lifetime) |  |  |  | Externalizing | 4 | 0 | 0 | 0 | 4 | 100% | 0.52 | 0.51 | 0.50 | 0.51 |
| Opioid (Past Year) |  |  |  | Externalizing | 4 | 0 | 0 | 0 | 4 | 100% | 0.51 | 0.52 | 0.50 | 0.51 |
| Opioid (Lifetime) |  |  |  | Externalizing | 4 | 0 | 0 | 0 | 4 | 100% | 0.53 | 0.53 | 0.50 | 0.52 |
| Sedative (Past Year) |  |  |  | Externalizing | 4 | 0 | 0 | 0 | 4 | 100% | 0.51 | 0.51 | 0.50 | 0.51 |
| Sedative (Lifetime) |  |  |  | Externalizing | 4 | 0 | 0 | 0 | 4 | 100% | 0.51 | 0.50 | 0.49 | 0.50 |
| Cocaine (Past Year) |  |  |  | Externalizing | 4 | 0 | 0 | 0 | 4 | 100% | 0.53 | 0.51 | 0.49 | 0.51 |
| Cocaine (Lifetime) |  |  |  | Externalizing | 4 | 0 | 0 | 0 | 4 | 100% | 0.58 | 0.53 | 0.55 | 0.55 |
| Hallucinogen (Past Year) |  |  |  | Externalizing | 4 | 0 | 0 | 0 | 4 | 100% | 0.50 | 0.50 | 0.50 | 0.50 |
| Hallucinogen (Lifetime) |  |  |  | Externalizing | 4 | 0 | 0 | 0 | 4 | 100% | 0.52 | 0.51 | 0.50 | 0.51 |
| Cannabis (Past Year) |  |  |  | Externalizing | 4 | 0 | 0 | 0 | 4 | 100% | 0.53 | 0.51 | 0.55 | 0.53 |
| Cannabis (Lifetime) |  |  |  | Externalizing | 4 | 0 | 0 | 0 | 4 | 100% | 0.55 | 0.54 | 0.49 | 0.53 |
| Gambling (Past Year) |  |  |  | Externalizing | 4 | 0 | 0 | 0 | 4 | 100% | 0.51 | 0.50 | X | 0.50 |
| Gambling (Lifetime) |  |  |  | Externalizing | 4 | 0 | 0 | 0 | 4 | 100% | 0.51 | 0.50 | X | 0.50 |
| Anti-Social PD (Lifetime) |  |  |  | Externalizing | 4 | 0 | 0 | 0 | 4 | 100% | 0.57 | 0.54 | X | 0.56 |
| Conduct Disorder (Lifetime) |  |  |  | Externalizing | 4 | 0 | 0 | 0 | 4 | 100% | 0.50 | 0.50 | X | 0.50 |
| Age < 20 or 44 > |  |  |  | Demographics | 3 | 0 | 0 | 0 | 3 | 100% | 0.33 | 0.40 | 0.50 | 0.41 |
| Age < 20 |  |  |  | Demographics | 3 | 0 | 0 | 0 | 3 | 100% | 0.49 | 0.53 | 0.50 | 0.51 |
| Age < 25 |  |  |  | Demographics | 3 | 0 | 0 | 0 | 3 | 100% | 0.55 | 0.55 | 0.54 | 0.54 |
| Age < 30 |  |  |  | Demographics | 3 | 0 | 0 | 0 | 3 | 100% | 0.54 | 0.55 | 0.50 | 0.53 |
| **Age < 35** | **** | **** | **** | **Demographics** | **3** | **0** | **0** | **0** | **3** | **100%** | **0.59** | **0.58** | **0.43** | **0.53** |
| **< HS Education** | **** | **** | **** | **Demographics** | **3** | **0** | **0** | **0** | **3** | **100%** | **0.55** | **0.52** | **0.50** | **0.52** |
| **Sexual Minority** | **** | **** | **** | **Demographics** | **3** | **0** | **0** | **0** | **3** | **67%** | **0.55** | **0.54** | X | **0.55** |
| **Female Sex at Birth** | **** | **** | **** | **Demographics** | **3** | **0** | **0** | **0** | **3** | **100%** | **0.57** | **0.55** | **0.62** | **0.58** |
| Male Sex at Birth | **** | **** | **** | Demographics | 3 | 0 | 0 | 0 | 3 | 100% | 0.43 | 0.45 | 0.38 | 0.42 |
| No Spouse | **** | **** | **** | Demographics | 3 | 0 | 0 | 0 | 3 | 100% | 0.56 | 0.58 | 0.71 | 0.62 |
| Veteran | **** | **** | **** | Demographics | 3 | 0 | 0 | 0 | 3 | 100% | 0.49 | 0.50 | 0.50 | 0.50 |
| Race: White | **** | **** | **** | Demographics | 3 | 0 | 0 | 0 | 3 | 100% | 0.43 | 0.48 | 0.41 | 0.44 |
| Race: Black | **** | **** | **** | Demographics | 3 | 0 | 0 | 0 | 3 | 100% | 0.50 | 0.50 | 0.58 | 0.53 |
| Race: American Indian | **** | **** | **** | Demographics | 3 | 0 | 0 | 0 | 3 | 100% | 0.52 | 0.51 | 0.55 | 0.52 |
| Race: Asian | **** | **** | **** | Demographics | 3 | 0 | 0 | 0 | 3 | 100% | 0.51 | 0.50 | 0.49 | 0.50 |
| Ethnicity: Latino | **** | **** | **** | Demographics | 3 | 0 | 0 | 0 | 3 | 100% | 0.55 | 0.51 | 0.48 | 0.51 |
| Ethnicity: Non-Latino | **** | **** | **** | Demographics | 3 | 0 | 0 | 0 | 3 | 100% | 0.45 | 0.50 | 0.53 | 0.49 |
| Psychosis/Schizophrenia | **** | **** | **** | Psychosis | 1 | 0 | 0 | 0 | 1 | 100% | 0.52 | 0.53 | 0.50 | 0.52 |
| **Mean** | | | | | | | | | | | **0.57** | **0.55** | **0.55** | **0.56** |

*Supporting Information* AUC = Area under the curve; DRS = Durham Risk Score; GAD = generalized anxiety disorder; HS = high school; NESARC = National Epidemiologic Survey on Alcohol and Related Conditions Study; NSSI = nonsuicidal self-injury; OCPD = obsessive-compulsive personality disorder; PD = personality disorder; PTSD = posttraumatic stress disorder; REHAB = Assessing and Reducing Post-Deployment Violence Risk Study; SITBI = Self-injurious thoughts and behaviors; SLE = stressful life event.

| **Table B** | | | |
| --- | --- | --- | --- |
| ***Measures used to Assess the Constructs Included in the Durham Risk Score.*** | | | |
| **Variable** | **NESARC** | **REHAB** | **VALOR** |
| Suicide Attempt - Lifetime | NESARC/AUDADIS: Item #S4AQ4A16 | SSQ: Actual Attempt Lifetime | MINI: Actual Attempt Lifetime |
| Suicidal Ideation - Lifetime ^a^ | NESARC/AUDADIS: Item #S4AQ4A17 or #S4AQ4A16 | SSQ: Ideation or Actual Attempt Lifetime or SCL-90: Item #15 | C-SSRS: Ideation or Actual Attempt Lifetime or PHQ Item #9 |
| Suicidal Ideation - Past Year | Not Assessed | SCL-90: Item #15 | C-SSRS: Ideation Past Year or PHQ Item #9 |
| NSSI - Lifetime ^b^ | NESARC/AUDADIS: Item #**W2S10Q1A30** | SSQ: NSSI Lifetime | **SITBI NSSI Lifetime** |
| Hospitalization - Lifetime | NESARC/AUDADIS: Item #S2CQ2A4, S3DQ2A4, S4AQ17A, S4CQ15A, S5Q18A, S6Q23, S7Q28, S8Q24B, S9Q16B, S2CQ2B4, or S3DQ2B4 | SSQ: Lifetime Hospitalization Item | Not Assessed |
| Hospitalization - Past Year | NESARC/AUDADIS: Item #S2CQ2B4 or S3DQ2B4 | Not Assessed | Not Assessed |
| Borderline PD - Lifetime ^b^ | NESARC/AUDADIS Item #**BPDDX** | Not Assessed | Not Assessed |
| Violence/Incarc. - Lifetime ^b^ | NESARC/AUDADIS: Item #S11AQ1A30, S11AQ1A32, S11AQ1A29, S11AQ1A28, S11AQ1A27, S11AQ1A26, S2BQ1A26, S3CQ12A2, S5Q7A5, or **W2S11Q6A** | DAST Item #14 or SSQ Violence Lifetime | Not Assessed |
| Mood Disorder - Lifetime | NESARC/AUDADIS: Item #MAJORDEPLIFE, DYSLIFE, NMANDXLIFE, or NHYPOMANLIFE | SCID-IV Mood Diagnosis Lifetime | PHQ Total Score = 10+ |
| SUD - Past Year | NESARC/AUDADIS: Item #ALCABDEP12DX, STIM12ABDEP, PAN12ABDEP, SED12ABDEP, TRAN12ABDEP, COC12ABDEP, SOL12ABDEP, HAL12ABDEP, MAR12ABDEP, HER12ABDEP, or OTHB12ABDEP | SCID-IV SUD Diagnosis Past Year | AUDIT Total Score = 16+ |
| PTSD - Past Month ^b^ | NESARC/AUDADIS: Item#**PLIPTSDK** and **SINPYPTSDK** | CAPS-IV - Current PTSD Dx | SCID-IV - PTSD Past Year |
| Weekly Binges - Past Year | NESARC/AUDADIS: Item #S2AQ4G, S2AQ5G, S2AQ6G, S2AQ7G, or S2AQ8E | AUDIT: Item #3 = 3+ | AUDIT: Item #3 = 3+ |
| Current Smoker | NESARC/AUDADIS: Item #SMOKER | SSQ: Smoker It | Not Assessed |
| Severe Sleep Problems - Past Year | Not Assessed | DTS Item #12 Severity = 4 | PHQ-9: Item #3 = 3 |
| Sexual Abuse/Assault ^b^ | NESARC/AUDADIS: Item #**W2S12Q5A4** | TLEQ Items #15, 16, 17, or 18 | LEC Item#8 = "Happened to me" |
| Child Physical Abuse ^b^ | NESARC/AUDADIS: Item #**W2S12Q5A5** | TLEQ Item #12 = 1+ | CTQ Physical Abuse Scale = 8+ |
| Lower Income | NESARC/AUDADIS: Item #S1Q11B: < $40,000 | Not Assessed | SSQ Income Item: < $35,000 |
| Unemployed | NESARC/AUDADIS: Item #S1Q235 | SSQ: Unemployment Item | SSQ: Unemployment Item |
| LGBTQ ^b^ | NESARC/AUDADIS: Item #**W2S14Q7** | Not Assessed | Not Assessed |
| Younger than 35 | NESARC/AUDADIS: Item #AGE | SSQ: Age Item | SSQ: Age Item |
| Female Sex | NESARC/AUDADIS: Item #SEX | SSQ: Sex Item | SSQ: Sex Item |
| Less than HS Education | NESARC/AUDADIS: Item #S1Q6A | SSQ: Education Item | Not Assessed |
| Poor Perceived Health | NESARC/AUDADIS: Item #S1Q16 = 4+ | Not Assessed | VR-12 Item#1 = 4+ |
| *Supporting Information:* NESARC = National Epidemiologic Survey on Alcohol and Related Conditions Study; REHAB = Assessing and Reducing Post-Deployment Violence Risk Study; VALOR = Veterans After-Discharge Longitudinal Registry Study; ^a^ Lifetime ideation was not directly assessed in sample 8; however, consistent with scoring procedures, participants with a history of attempts or current ideation were scored as also having lifetime ideation. ^b^ Items in bold were assessed at the time of the follow-up interview. All other items were assessed at the time of the baseline interview. LGBTQ = Lesbian-Gay-Bisexual-Transgendered-Questioning; HS = High School; NESARC = National Epidemiologic Survey on Alcohol and Related Conditions; C-SSRS = Columbia Suicide Severity Rating Scale; SITBI = Self-Injurious Thoughts and Behaviors Interview; SCID-IV = Structured Clinical Interview for DSM-IV; CAPS-IV = Clinician-Administered PTSD Scale for DSM-IV; MINI = Mini International Neuropsychiatric Interview; AUDADIS = Alcohol Use Disorder and Associated Disabilities Interview Schedule; AUDIT = Alcohol Use Disorders Identification Test; DAST = Drug Abuse Screening Test; DTS = Davidson Trauma Scale; PHQ-9 = Patient Health Questionnaire-9; TLEQ = Traumatic Life Events Questionnaire; CTQ = Childhood Trauma Questionnaire; VR-12 = Veterans Rand 12-Item Health Survey; LEC = Life Events Checklist; SCL-90 = Symptom Checklist-90; SSQ = study-specific questionnaire. | | | |

**Table C**

***Distribution, Rates of Suicide Attempts, Odds, and Predicted Probabilities by Risk Group Status in Total Sample (N=35,654).***

| **Durham Risk Score Total** | **Risk Group** | **% of Sample** | **Total # of Participants** | **# of Participants who Attempted Suicide** | **% of Participants who Attempted Suicide** | **Risk Ratio** | **Odds** | **Odds Ratio** | **Predicted Probability** |
| --- | --- | --- | --- | --- | --- | --- | --- | --- | --- |
| 0 - 2 | Lowest Risk | 44.6% | 15,903 | 4 | 0.03% | 1.0 | 0.0003 | 1.0 | 0.03% |
| 3 - 5 | Low Risk | 40.6% | 14,475 | 48 | 0.3% | 13.2 | 0.003 | 13.2 | 0.3% |
| 6 - 8 | Moderate Risk | 9.9% | 3,545 | 70 | 2% | 78.5 | 0.02 | 80.1 | 2% |
| 9 - 11 | High Risk | 3.3% | 1,176 | 69 | 6% | 233.3 | 0.06 | 247.7 | 6% |
| 12 - 14 | Very High Risk | 1.0% | 362 | 45 | 12% | 494.2 | 0.14 | 564.2 | 12% |
| 15 - 30 | Highest Risk | 0.5% | 193 | 52 | 27% | 1071.2 | 0.37 | 1,465.9 | 27% |
|  |  |  | 35,654 | 288 | 0.81% |  |  |  |  |

*Supporting information:* # = number; % = percent.

**Table D**

***Association between Area under the Curve (AUC) Values and Number of Items Assessed Across Samples.***

| **Sample Name** | **Number of Durham Risk Score (DRS) Items Assessed in Sample** | **Area Under the Curve (AUC)** |
| --- | --- | --- |
| NESARC 1 | 21 | 0.93 |
| NESARC 2 | 21 | 0.89 |
| REHAB | 18 | 0.88 |
| NESARC 3 | 21 | 0.92 |
| NESARC 4 | 21 | 0.92 |
| VALOR | 15 | 0.82 |
| NESARC 1: Sensitivity Analysis to Assess Impact of Cross-Sectional Items ^a^ | 15 | 0.86 |
| NESARC 2: Sensitivity Analysis to Assess Impact of Cross-Sectional Items ^a^ | 15 | 0.79 |
| NESARC 3: Sensitivity Analysis to Assess Impact of Cross-Sectional Items ^a^ | 15 | 0.87 |
| NESARC 4: Sensitivity Analysis to Assess Impact of Cross-Sectional Items ^a^ | 15 | 0.85 |
| *Supporting Information:* NESARC = National Epidemiologic Survey on Alcohol and Related Conditions Study; REHAB = Assessing and Reducing Post-Deployment Violence Risk Study; VALOR = Veterans After-Discharge Longitudinal Registry Study; ^a^ The fifteen items included in the NESARC sensitivity analyses designed to assess the impact of cross-sectionally assessed items included only items that were assessed at the baseline assessment, including suicide attempt, past year hospitalization, lifetime hospitalization, lifetime ideation, poor perceived health, low income, unemployment, lifetime mood disorder, weekly binge drinking, current smoker, current substance use disorder, younger than 35 years of age, less than high school education, female, and lifetime history of violence and incarceration. Please note that item #W2S11Q6A (which was assessed at wave 2) was also removed from the calculation of lifetime history of violence and incarceration for these analyses. Thus, all variables used in the calculation of the DRS in these sensivity analyses were assessed at the time of the wave 1 interview and prior to the occurence of any prospective suicide attempts that occurred between waves 1 and 2. | | |

**Table E**

| ***Summary of Logistic Regression Conducted in the Combined NESARC 1 & 2 Development Samples (N = 17,397).*** | | | | | | |
| --- | --- | --- | --- | --- | --- | --- |
| Variable | **B** | **SE** | **OR** | **Lower 95% CI for OR** | **Upper 95% CI for OR** | ***p*** |
| Suicide Attempt (Lifetime) | 0.53 | 0.33 | 1.69 | 0.88 | 3.26 | 0.12 |
| Hospitalization (Past Year) | 0.02 | 0.56 | 1.02 | 0.34 | 3.07 | 0.98 |
| **Hospitalization (Lifetime)** | **0.95** | **0.29** | **2.58** | **1.47** | **4.50** | **0.001** |
| **Nonsuicidal Self-Injury (Lifetime)** | **1.26** | **0.26** | **3.52** | **2.12** | **5.85** | **< .001** |
| Suicidal Ideation (Lifetime) | 0.40 | 0.34 | 1.50 | 0.77 | 2.93 | 0.24 |
| **Borderline Personality Disorder (Lifetime)** | **1.77** | **0.24** | **5.87** | **3.65** | **9.43** | **< .001** |
| **Unemployed** | **0.53** | **0.25** | **1.70** | **1.04** | **2.76** | **0.03** |
| **Poor Perceived Health (Current)** | **0.73** | **0.23** | **2.07** | **1.32** | **3.25** | **0.001** |
| Lower Income | 0.17 | 0.23 | 1.18 | 0.75 | 1.87 | 0.47 |
| **Childhood Physical Abuse (Lifetime)** | **0.55** | **0.27** | **1.73** | **1.02** | **2.93** | **0.04** |
| Sexual Abuse or Assault (Lifetime) | 0.43 | 0.26 | 1.54 | 0.94 | 2.54 | 0.09 |
| **Posttraumatic Stress Disorder (Current)** | **0.50** | **0.25** | **1.66** | **1.02** | **2.70** | **0.04** |
| Mood Disorder (Lifetime) | -0.20 | 0.29 | 0.82 | 0.46 | 1.44 | 0.48 |
| History of Violence or Incarceration (Lifetime) | -0.30 | 0.24 | 0.74 | 0.46 | 1.20 | 0.22 |
| Weekly Binge Drinker (Current) | -0.17 | 0.39 | 0.84 | 0.40 | 1.79 | 0.66 |
| Current Smoker | 0.17 | 0.22 | 1.19 | 0.77 | 1.83 | 0.44 |
| Substance Use Disorder (Current) | 0.34 | 0.30 | 1.41 | 0.78 | 2.54 | 0.26 |
| **Younger than 35** | **0.44** | **0.22** | **1.55** | **1.00** | **2.41** | **0.048** |
| Less than High School Education | 0.05 | 0.25 | 1.06 | 0.65 | 1.73 | 0.83 |
| **Lesbian-Gay-Bisexual-Transgendered-Questioning** | **1.16** | **0.33** | **3.18** | **1.66** | **6.11** | **0.001** |
| Female at Birth | 0.20 | 0.24 | 1.22 | 0.76 | 1.97 | 0.41 |
| Constant | -6.80 | 0.28 |  |  |  | < .001 |

*Supporting Information:* Variables that are statistically signicant at *p* < 0.05 are shown in bold; B = beta; SE = standard error; OR = odds ratio; CI = confidence interval.

**Table F**

| ***Items used to Calculate the SAD PERSONS Score in the NESARC Study.*** | | |
| --- | --- | --- |
| **Variable** | | **Item(s) Used to Calculate Variable** |
| **S** | Male **S**ex | NESARC/AUDADIS: Item #SEX |
| **A** | **A**ge < 20 or > 44 | NESARC/AUDADIS: Item #AGE |
| **D** | **D**epression | NESARC/AUDADIS: Item #MAJORDEPLIFE |
| **P** | **P**revious Attempt | NESARC/AUDADIS: Item #S4AQ4A16 |
| **E** | **E**thanol Abuse | NESARC/AUDADIS: Item #ALCABDEP12DX or ALCABDEPP12DX |
| **R** | Loss of **R**ational Thinking | NESARC/AUDADIS: Item # S13Q6C, NMANDXLIFE, or NHYPOMANLIFE |
| **S** | **S**ocial Supports Lacking | NESARC/AUDADIS: Item #S1Q231, S1Q234, S1Q235, or S1Q238 |
| **O** | **O**rganized Plan | NESARC/AUDADIS: Item #S4AQ4A17 |
| **N** | **N**o Spouse | NESARC/AUDADIS: Item #MARITAL |
| **S** | **S**ickness | NESARC/AUDADIS: Item #S13Q6B1, S13Q6B2, S13Q6B3, S13Q6B4, S13Q6B5, S13Q6B6, S13Q6B7, S13Q6B8, S13Q6B9, S13Q6B10, or S13Q6B11 |
| *Supporting Information:* NESARC = National Epidemiologic Survey on Alcohol and Related Conditions Study; AUDADIS = Alcohol Use Disorder and Associated Disabilities Interview Schedule; AUDIT = Alcohol Use Disorders Identification Test. | | |

**Fig A**

***Association between Total Empirical Evidence Score and Mean AUC Value Across the Development Samples.***

***
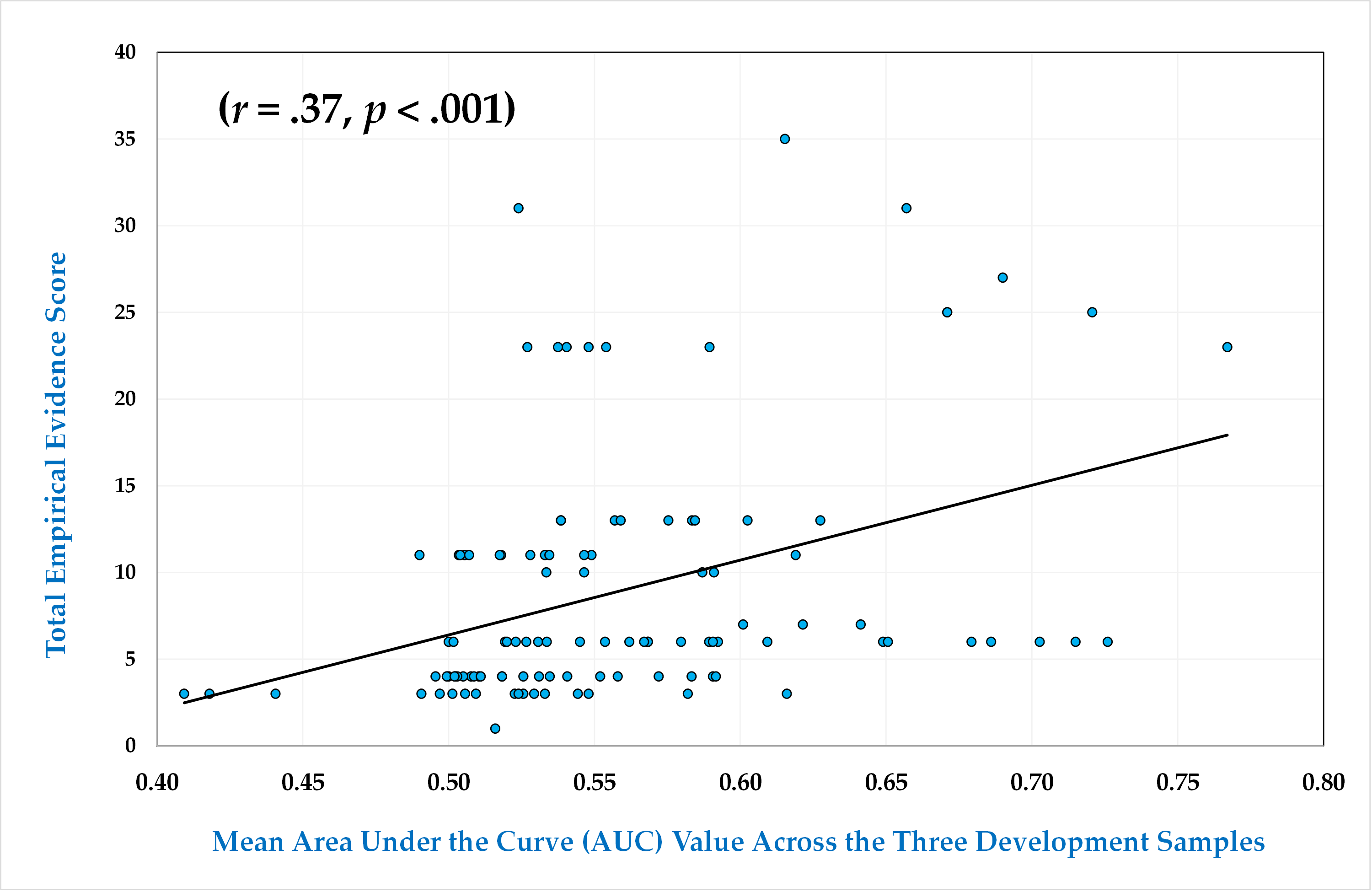
***

*Supporting Information:* Vertical axis = Total Empirical Evidence Score; Horizontal axis = Mean AUC value across the three development samples. AUC = area under the curve; *r* = correlation; total empirical evidence scores and mean bivariate AUC values were obtained from S1 Table A.

**Fig B**

***Distribution of Durham Risk Scores.***

***
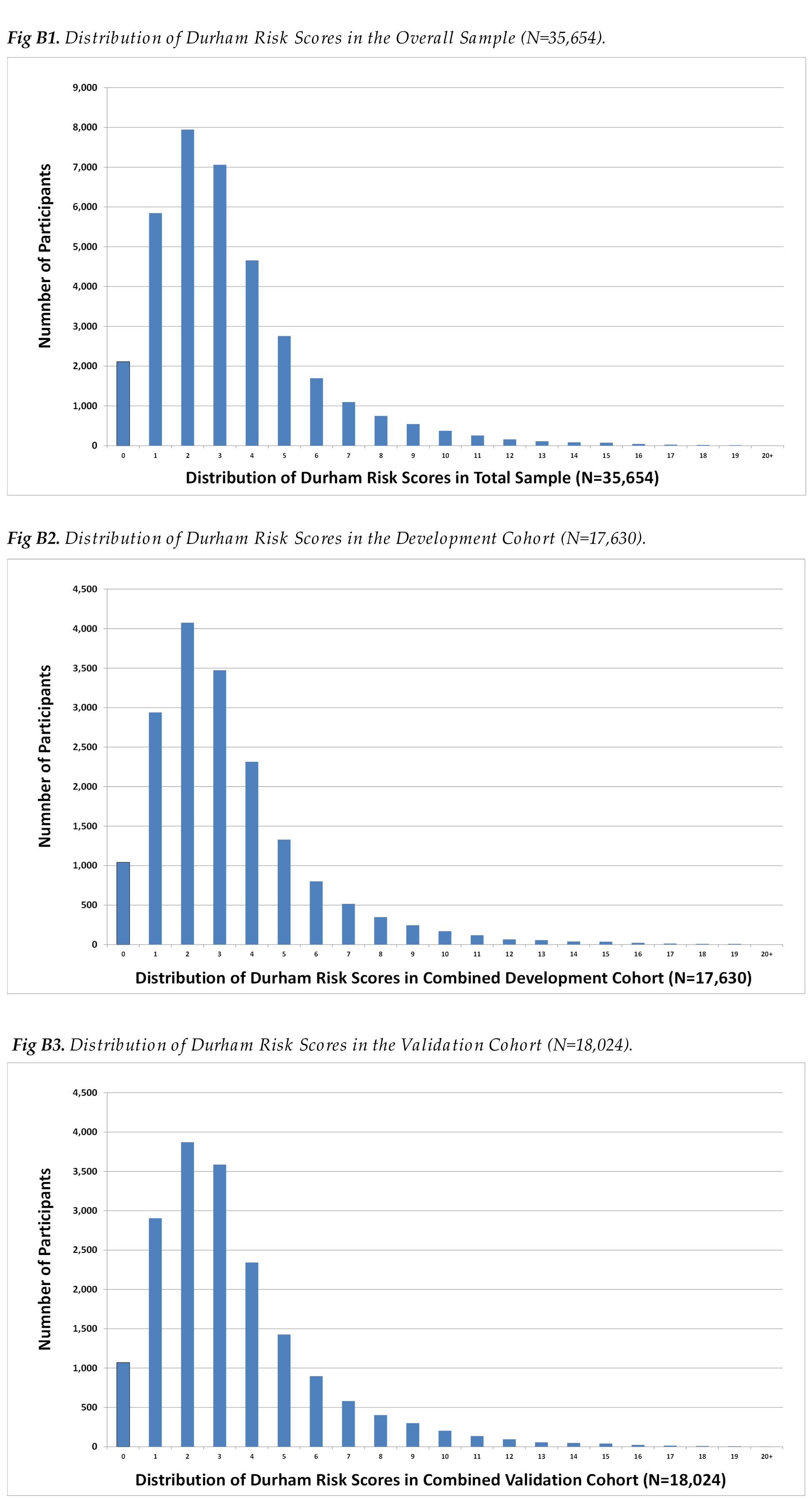
***

*Supporting information:* Vertical axis = number of participants. Horizontal axis = Durham Risk Scores.

**Fig C**

***Distribution of Durham Risk Scores among Participants who Attempted Suicide during Follow-Up (N=288).***

***
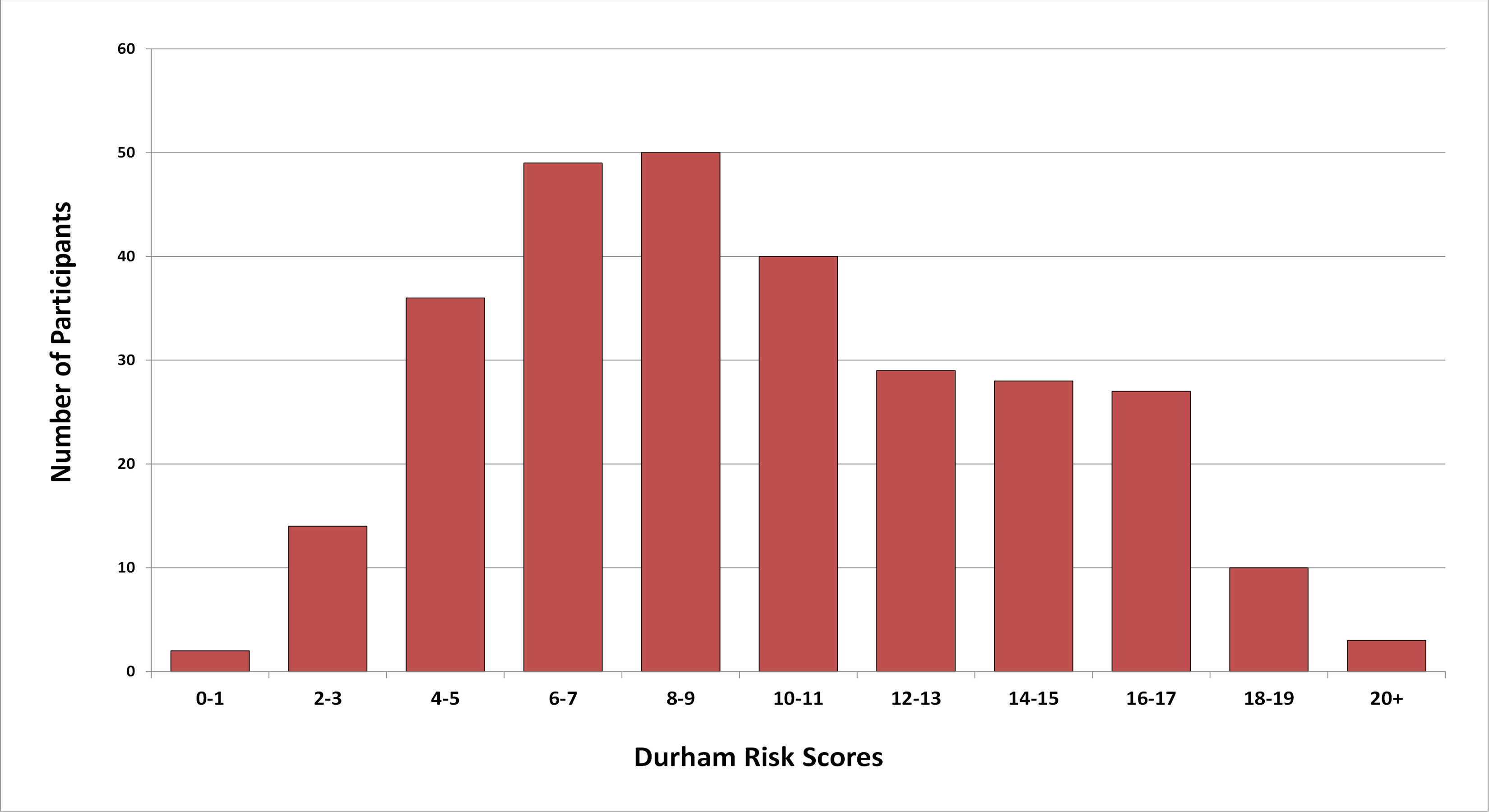
***

*Supporting information:* Vertical axis = number of participants. Horizontal axis = Durham Risk Scores.

**Fig D**

***Association between Number of Items and Area Under the Curve Values.***

***
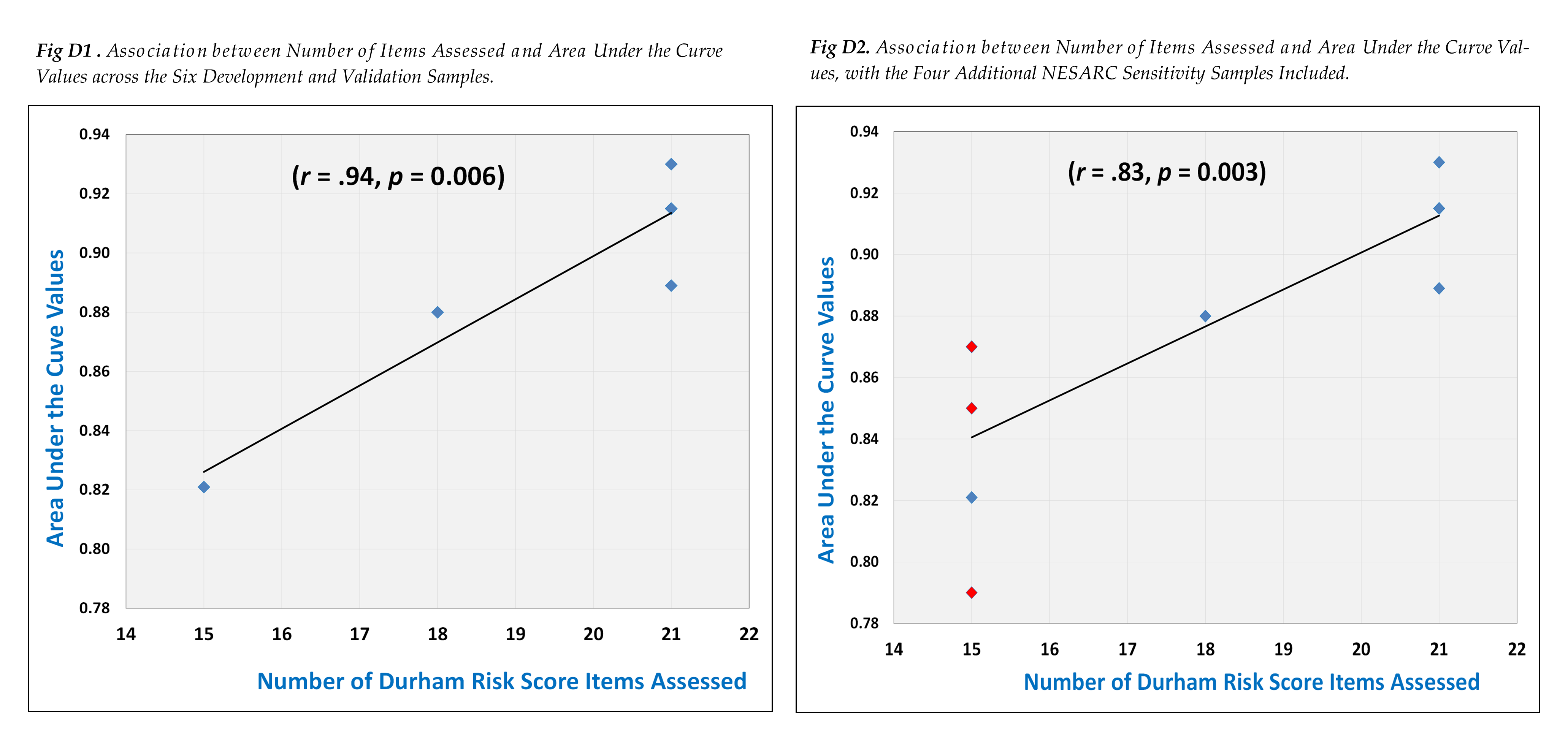
***

***Supporting Information.*** Blue diamonds correspond to the values for NESARC 1, NESARC 2, REHAB, NESARC 3, NESARC 4, and VALOR from S1 Table D, whereas red diamonds correspond to the values for the four NESARC sensitivity samples from S1 Table D; NESARC = National Epidemiologic Survey on Alcohol and Related Conditions Study; REHAB = Assessing and Reducing Post-Deployment Violence Risk Study; VALOR = Veterans After-Discharge Longitudinal Registry Study; r = correlation.
